# Astragaloside IV promotes pharmacological effect of *Descurainia sophia* seeds on isoproterenol-induced cardiomyopathy in rats by synergistically modulating the myosin motor

**DOI:** 10.3389/fphar.2022.939483

**Published:** 2022-08-11

**Authors:** Xingkai Liu, Qian Chen, Xuming Ji, Wanchen Yu, Tong Wang, Juanjuan Han, Shumu Li, Jianan Liu, Fangang Zeng, Yao Zhao, Yanyan Zhang, Qun Luo, Shijun Wang, Fuyi Wang

**Affiliations:** ^1^ Beijing National Laboratory for Molecular Sciences, CAS Research/Education Center for Excellence in Molecular Sciences, National Centre for Mass Spectrometry in Beijing, CAS Key Laboratory of Analytical Chemistry for Living Biosystems, Institute of Chemistry, Chinese Academy of Sciences, Beijing, China; ^2^ University of Chinese Academy of Sciences, Beijing, China; ^3^ College of Traditional Chinese Medicine, Shandong University of Traditional Chinese Medicine, Jinan, China; ^4^ Academy of Chinese Medical Science, School of Basic Medical Science, Zhejiang Chinese Medical University, Hangzhou, China; ^5^ School of Environment and Natural Resources, Renmin University of China, Beijing, China

**Keywords:** astragaloside IV, cardiomyopathy, myosin, quantitative proteomics, *Descurainia sophia* seed, traditional Chinese medicine

## Abstract

*Descurainia sophia* seeds (DS), *Astragalus mongholicus* (AM), and their formulas are widely used to treat heart failure caused by various cardiac diseases in traditional Chinese medicine practice. However, the molecular mechanism of action of DS and AM has not been completely understood. Herein, we first used mass spectrometry coupled to UPLC to characterize the chemical components of DS and AM decoctions, then applied MS-based quantitative proteomic analysis to profile protein expression in the heart of rats with isoproterenol-induced cardiomyopathy (ISO-iCM) before and after treated with DS alone or combined with AM, astragaloside IV (AS4), calycosin-7-glucoside (C7G), and Astragalus polysaccharides (APS) from AM. We demonstrated for the first time that DS decoction alone could reverse the most of differentially expressed proteins in the heart of the rats with ISO-iCM, including the commonly recognized biomarkers natriuretic peptides (NPPA) of cardiomyopathy and sarcomeric myosin light chain 4 (MYL4), relieving ISO-iCM in rats, but AM did not pronouncedly improve the pharmacological efficiency of DS. Significantly, we revealed that AS4 remarkably promoted the pharmacological potency of DS by complementarily reversing myosin motor MYH6/7, and further downregulating NPPA and MYL4. In contrast, APS reduced the efficiency of DS due to upregulating NPPA and MYL4. These findings not only provide novel insights to better understanding in the combination principle of traditional Chinese medicine but also highlight the power of mass spectrometric proteomics strategy combined with conventional pathological approaches for the traditional medicine research.

## Introduction

Cardiomyopathy is one of the leading causes of heart failure, and dilated cardiomyopathy (DCM) and hypertrophic cardiomyopathy (HCM) are the two most common cardiomyopathies (Yamada and Nomura, 2021). HCM is a genetic disease, to which hundreds of mutations in genes encoding cardiac myosin and sarcomere-related proteins are linked (Lehman et al., 2022). However, various factors, such as ischemia, viral infection, and alcohol toxicity, could cause DCM, though the variants in genes encoding sarcomeric proteins, for example, myosin heavy chain-β and -α (*MYH7* and *MYH6*), and the myosin light chain *MYL2* were also implicated in DCM (Kamisago et al., 2000; Yuan et al., 2018). Sarcomeric myosins act as the molecular accelerator/brake, modulating cardiac contractility (Daniels et al., 2021). The inherited HCM and DCM are both attributable to pathogenic variants in the myosin motor, which alters the proportion of the myosin accelerator and brake (Daniels et al., 2021). Therefore, directly targeting sarcomere in genetic cardiomyopathies has been thought to be a root-eradicating strategy for the treatment of these diseases without side effects on other organs. Both myosin activators and inhibitors have been developed and shown great promise for therapeutics of HCM and/or DCM (Lehman et al., 2022). For example, danicamtiv, a promising cardiac myosin activator, also known as MYK-491, was reported to selectively promote cardiac actomyosin activity by increasing myosin head availability and phosphate release rates (Shen et al., 2021), reversing respective contractile disordered in engineered heart tissue models of HCM and DCM (Halder et al., 2021). In contrast, mavacamten, a myosin inhibitor, also known as MYK-461, was shown to reduce sarcomere contractility in HCM by binding to myosin and decreasing phosphate release rates (Anderson et al., 2018; Lehman et al., 2022).

Traditional Chinese medicines (TCMs) have been used for long time to treat cardiac diseases including DCM and HCM (Yu et al., 2019; Li et al., 2020; Tan et al., 2020). Among them, *Descurainia sophia* seeds (DS; Brassicaceae; *Descurainia sophia* (L.) Webb ex Prantl. seed) and *Astragalus mongholicus Bunge* (AM; Leguminosae*; Astragalus mongholicus Bunge* radix) and their combination are widely used to treat heart failure caused by various cardiac diseases (Ma et al., 1998; Chen et al., 2006a; Zhou et al., 2016; Yu et al., 2019; Liu et al., 2020; Huang et al., 2021; Yu et al., 2021). Quercetin and kaempferol were identified by a network pharmacology study, to be the main active components in the DS–AM formula. They were believed to relieve heart injury by regulating PI3K-Akt, VEGF, and erbB signaling pathways, and insulin resistance (Yu et al., 2021). Quercetin is one of the active flavone components in many Chinese herbal medicines. Our pharmacological studies showed that DS decoction could alleviate heart failure by inhibiting excessive activation of the neuroendocrine system, strengthening myocardial contractility, and reducing end-diastolic volume and pressure to improve cardiac hemodynamics. However, quercetin alone produced less pharmacological effect than DS used in the whole (unpublished data). In TCM practices AM is believed to be an important Qi tonic medicine and can replenish Qi and Yang, promoting diuresis and detumescence and enhancing myocardial muscle strength. More studies on the mechanism of action of AM, in particular the three major active components astragaloside IV (AS4) (Liu et al., 2018; Lin et al., 2019; Tan et al., 2020; Zang et al., 2020), calycosin-7-glucoside (C7G), and Astragalus polysaccharides (APS), have also been reported. The major bioactive components astragalosides, in particular astragaloside IV (AS4), from AM have been extensively studied (Zhang et al., 2015; Xu et al., 2016; Tan et al., 2020; Du et al., 2022) were shown to exert a beneficial effect on myocardial lesion by regulating ATP consumption and preserving intracellular Ca^2+^ homeostasis (Chen et al., 2006b). Wang et al. reported that AS4 reduced cardiac hypertrophy induced by aortic banding surgery in mice *via* inactivating the TBK1/PI3K/AKT signaling pathway (Liu et al., 2018). Zhang et al. (2015)demonstrated that AS4 released ISO-induced cardiac hypertrophy *via* regulating NF-κB/PGC-1α signaling and improved the ISO-induced vascular dysfuntion by regulating eNOS uncoupling-mediated oxidative stress and retarding ROS-NF-κB signaling (Xu et al., 2016). Xiao et el. showed that AS4 acting as an antioxidant relieved doxorubicin-induced cardiomyopathy *in vitro* and *in vivo* by suppressing NADPH oxidase expression (Lin et al., 2019). AS4 was also revealed to ameliorate isoprenaline-induced cardiac fibrosis in mice by modulating gut microbiota and fecal metabolites (Du et al., 2022). Calycosin is another major active component in AM and was shown to attenuate oxidative stress-induced cardiomyocyte apoptosis by activating estrogen receptors and promoting AKT phosphorylation (Liu et al., 2016). Very recently, calysosin was reported to act as a PI3K activator to ameliorate inflammation and fibrosis in heart failure *via* the AKT-IKK-STAT3 axis (Wang et al., 2022). Astragalus polysaccharide was revealed to be able to inhibit diabetic cardiomyopathy in hamsters by suppressing chymase activation (Chen et al., 2010a; Chen et al., 2010b). However, despite DS and AM and their active components have been extensively studied, the molecular mechanism of action of them has not been completely understood. For instance, how DS and AM or their active components regulate PI3K/AKT phosphorylation signaling pathway? How DS and AM or their active components regulate ATP consumption and maintain intracellular Ca^2+^ homeostasis?

To address these issues, in the present work, we applied a mass spectrometry (MS)-based quantitative proteomics strategy to explore the mechanism of action of DS combined with AM or the major active components, astragaloside IV and calysosin-7-glucoside, of AM. The studies were performed on rats with isoproterenol-induced cardiomyopathy (ISO-iCM) (Xie et al., 2015). With quantitatively comparing the protein expression levels in the heart of the sacrificed control, model, and treated rats, we demonstrated for the first time that DS decoction ameliorates ISO-iDM in rats by significantly downregulating the typical biomarkers of cardiomyopathy, including natriuretic peptides A (NPPA) and myosin light chain 4 (MYL4) (Tripathi et al., 2017). More importantly, we provided molecular evidence for that AM did not improve the pharmacological effect of DS on ISO-iDM, but astragaloside IV (AS4) in AM remarkably promoted the pharmacological efficacy of DS by reversing the expression of the sarcomeric motor myosin heavy chain 7 (MYH7) (Daniels et al., 2021), and further downregulating NPPA and MYL4. The combination of DS with AS4 interestingly showed a similar beneficial effect on cardiac contractibility to the myosin inhibitor mavacamten under clinical trial (Anderson et al., 2018; Lehman et al., 2022).

## Materials and method

### Reagents

*Descurainia sophia* seeds (DS; Brassicaceae; *Descurainia Sophia* (L.) Webb ex Prantl. seed) and *Astragalus mongholicus Bunge* (AM; *Leguminosae; Astragalus mongholicus Bunge* radix) were purchased from Baiweitang Decoction Pieces Co. (China). The two plant materials were identified by the affiliated hospital of Shandong University of Traditional Chinese Medicine, with Specimen Nos. SUTCM/PHAR/HRB/21/05/17 and SUTCM/PHAR/HRB/20/03/18, respectively. *Astragalus* polysaccharides (P/N: 7105MC, w/w 99%) was provided by Medicass Biological Products Co. (China). Astragaloside IV and calycosin-7-glucoside were obtained from Nature Standard Technical Service Co. (China). The low protein binding microcentrifuge tube was purchased from Thermo Fisher Scientific Ltd. (United States). Sodium chloride obtained from Sinopharm Chemical Reagent Co., Tris base from BioDee (China), and Tween-20 from Solarbio (China) were used to prepare TBST buffer. HPLC grade water (Fisher Chemical) was used throughout all experiments.

### Animals and models

In primary experiments, we did not find significant difference in the pharmacological efficiency of DS and AM and various combinations between male and female rats with isoproterenol-induced cardiomyopathy (ISO-iCM). Therefore, in the present work, we selected 1:1 of male and female rats to establish of ISO-iCM models. To this end, 77 specific-pathogen-free, Sprague–Dawley rats (half male and half female), weighing 260 ± 20 g, were purchased from Beijing Vital River Laboratory Animal Technology Co. Ltd (Beijing, China), with a certification number of SCXK (Jing) 2012-0001. The rats were acclimated for 3 days in the animal facility of the Shandong University of Traditional Chinese Medicine and then randomly divided into seven groups, designated as control group (CG), model group (MG), and treated groups with DS decoction alone (DS), DS combined with AM (DSAM), astragaloside IV (DSAS4), calycosin-7-glucoside (DSC7G), or Astragalus polysaccharides (DSAPS) decoction, respectively, with 11 rats in each group. Isoproterenol was used to induce cardiomyopathy, following the procedure reported previously (Xie et al., 2015). In brief, isoproterenol (3 mg kg^−1^•d^−1^) was subcutaneously injected into the scapular region of each rat for 10 days. After 2 weeks of observation, an endotracheal tube was implanted in each rat by using an endotracheal intubation device. The catheter was retained for 7 days prior to further experiments. Notably, the endotracheal tube was used to establish the models with the Upper Energizer Stage patterns (Code: SG70 in the International Statistical Classification of Diseases and Related Health Problems (ICD-11)) with the fluid disturbance pattern (ICD Code: SF11), accompanied with isoproterenol stimulus. However, in the present work, the pharmacological benefits of all formulas were evaluated only based on the heart of the models. All animal experiments were approved by the Ethics Committee of Shandong University of Traditional Chinese Medicine and carried out in accordance with the Laboratory Animal-Guideline for Ethical Review of Animal Welfare (GB/T 35892-2018).

### Preparation and administration of drugs

The DS and AM decoctions were prepared following the traditional clinical medication method described in Chinese Pharmacopoeia (2010 Edition, p283 and p313). In brief, an aliquot of purchased DS seeds and AM slices were individually put into a casserole, followed by the addition of 10-fold tap water to obtain a final concentration of 0.18 g/ml for DS and 0.54 g/ml for AM, and stilled at room temperature for 0.5 h and then boiled for 1 h. The raw decoction was filtered, and the filtrate was collected. The boiling and filtration were repeated three times, and the filtrates were merged. The merged decoctions were evaporated to the initial values, of which the final concentrations of total extracts were 2.18 mg/ml (DS) and 6.53 mg/ml (AM), respectively. The mixed decoction of DS and AM (1:3, w/w) was prepared following the procedure described earlier, of the final concentration of total extract was 8.73 mg/ML. Aliquots of AS4, C7G, and APS were individually dissolved in 0.5% sodium carboxymethyl cellulose solution to prepare the suspensions of 3 mg/ml for AS4, 1.5 mg/ml for C7G, and 141 mg/ml for APS, which were stored at 4°C before use.

After pathological assessments of all rats with ISO-iDM, DS decoction and the various combinations were administrated. Based on the Chinese Pharmacopoeia (2010 Edition), 10 ml kg^−1^•d^−1^ of DS decoction (containing 21.8 mg of extract in total) was administrated to each rat of the DS group, 10 ml kg^−1^•d^−1^ of mixed decoction of DS and AM (containing 87.3 mg of extract in total) to each rat of the DSAM group, and 10 ml g•kg^−1^•d^−1^ of DS decoction plus 50 mg kg^−1^•d^−1^ of AS4, 30 mg kg^−1^•d^−1^ of C7G, or 41 mg kg^−1^•d^−1^ of APS, were administrated to each rat of the DSAS4, DSC7G, or DSAPS group, respectively, by gavage once a day for 14 days. While the rats in the control and model groups were given normal saline at the same dose.

### Characterization of components in *Descurainia sophia* seeds and *Astragalus mongholicus*

#### Preparation of *Descurainia sophia* seeds (DS) and *Astragalus mongholicus* (AM) samples

Aliquot (100 μL) of DS or AM decoction prepared as described earlier was diluted by 900 μL method chloroform (3:2) mixture, and then 200 μL deionized water was added. The resulting mixture was centrifuged at 15,000 g, 4°C for 15 min. The up-layer was then transferred to a new tube and dried in a vacuum centrifuge. The residue was re-dissolved by 150 μL 80% MeOH/H_2_O containing 0.25% formic acid (FA) and 80% MeOH/H_2_O containing 20 mM ammonium acetate for positive and negative ESI-MS/MS analyses, respectively.

#### UPLC–MS/MS

The UPLC–MS/MS characterization of components in DS and AM decoctions was performed on a SYNAPT XS mass spectrometer coupled with the Waters ACQUITY Class I UPLC PLUS and an ACQUITY UPLC^®^ HSS T3 column (i.d. 1.8 μm, 100 mm × 2.1 mm) (Waters, United States). The mobile phases were water containing 0.25% FA (A) and methanol containing 0.25% FA (B) for positive mode, and water containing 20 mM ammonium acetate (A) and methanol containing 20 mM ammonium (B) for negative mode, respectively. For both positive and negative detections, the mobile phase B started at 2% and increased to 5% at 1 min, and further increase to 45% at 7 min, to 85% at 10 min, and to 95% at 17 min, with a flow rate of 0.17 ml/min.

The mass spectrum data were collected in HDMS_e_ mode, and the detection parameters were set as follows: acquiring mass range from 95-1000 Da under sensitivity acquisition mode; scan time 1.5 s; low transfer collision energy 15 V; and high ramp transfer collision energy 60–80 V. The lock-mass compound was leucine enkephalin (0.2 ng/ml).

#### Data analysis

Progenesis QI was used to interpret MS raw data. The mixture of DS and AM (1:1) decoction was used to align the chromatographic peaks for each mass spectrum file. For positive ion detection, the adduct ions including [M + H]^+^, [M + Na]^+^, [M + K]^+^, [M-H_2_O + H]^+^, and [M+2H]^2+^ were selected. The adduct ions [M-2H]^2-^, [M-H_2_O-H]^-^, [M-H]^-^, and [M + Cl]^-^ were selected for the negative mode. The compounds which have the highest abundance in the blank group or Anova(p) value larger than 0.0001 were filtered out for future analysis. KEGG, Human Metabolome Database, BroadPharm, Food and Agriculture Organization, xPharm, AQ BioPharma, AxisPharm, BLDpharm, abcr, and PubMed metabolite databases, and Nature Chemical Biology, Nature Chemistry, Nature Communications, and Springer Nature databases, were used to identify compounds detected by MS. The precursor tolerances and fragmentation tolerances were set 10 and 5 ppm, respectively. After identification, the compounds with Anova(p) value <0.0001, abundance >5000, and abundance ratio between AM and DS > 25 or <0.04 were considered as the main contents for AM and DS, respectively.

### Echocardiography

After the intervention described earlier, rats in all groups were examined by high-frequency echocardiography with a pb207 probe (20 MHz). The left ventricular ejection fraction (LVEF) and the left ventricular short-axis shortening fraction (LVFS) were measured by ultrasonic measurement.

### Determination of cardiac index and left ventricular mass index

Rats were weighed, then injected intraperitoneally with 10% chloral hydrate (3 ml/kg), anesthetized, and fixed. Blood was collected from the main abdominal vein and placed in an anticoagulant tube at room temperature for 2 h, centrifuged at 4°C, and 4,000 g for 10 min. The supernatant was stored at −80°C for determination of the creatine kinase (CK) level by enzyme-linked immunosorbent assay kit (Jiancheng Bioengineering Institute, China). Then, the rats were sacrificed to remove hearts, which washed off the residual blood with 0.9% ice normal saline, dried with filter paper, and weighed. The left atrium was cut off along the coronary sulcus, and the right ventricle was removed along the interventricular sulcus. The left ventricular was weighed, and the cardiac index (cardiac weight/body weight, HWI) and left ventricular weight index (left ventricular weight/body weight, LVWI) were thereby calculated.

### Histopathological assays

The left ventricular myocardial tissue of each rat was fixed with 10% paraformaldehyde for 24 h and then embedded in paraffin. The tissue sections were stained with hematoxylin–eosin and Masson (Wuhan Google Biotechnology, China) for pathological assays on a Nikon Eclipse optical microscope (Nikon, Japan). The collagen fiber volume fraction (CVF) of Masson’s stained sections was calculated by the ipp6.0 image analysis system (Media Cybernetics).

### Quantitative proteomics analysis

**FIGURE 1:**
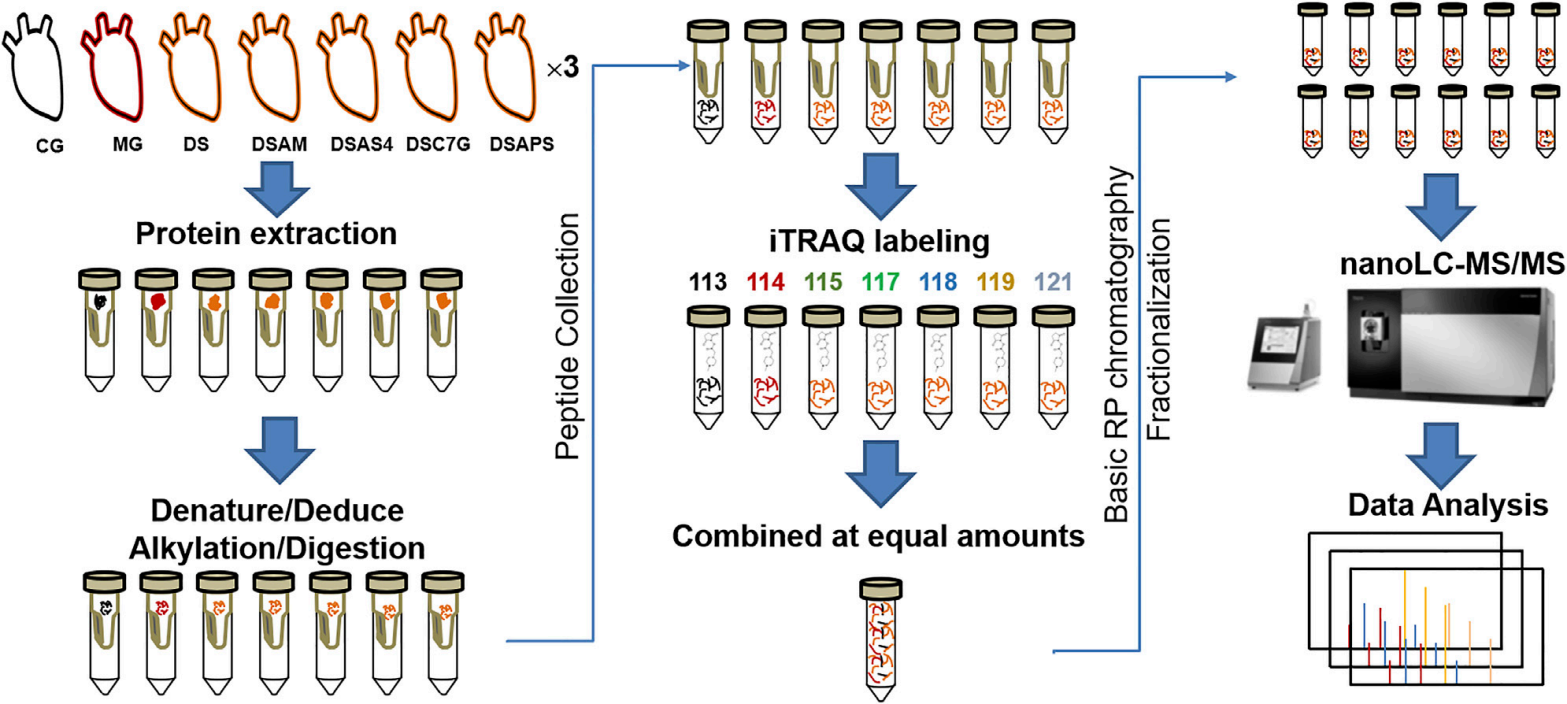
Workflow of quantitative proteomics analysis. The heart tissues of rats from seven groups are designated as the control group (CM), ISO-iCM model group (MG), and treated groups with *Descurainia sophia* seed decoction alone (DS), and combined with *Astragalus mongholicus* (DSAM), astragaloside IV (DSAS4), astragalus calycosin-7-glucoside (DSC7G), or astragalus polysaccharides (DSAPS), respectively.

The workflow of quantitative proteomics analysis is shown in Figure 1, and the detailed procedures are described as follows.

#### Protein extraction and digestion

RIPA lysis buffer (Beyotime, China) was added to ca. 0.5 g of rat heart tissue at 100 μL:10 mg ratio, the tissue was then lysed for 30 s by a homogenate machine (JXFSTPRP-24, Shanghai Jingxin Industrial Development Co., Ltd.) at 50% pulse for 5 times. The raw protein extract was centrifuged at 12,000 g for 15 min, and then the supernatant was transferred to a PE tube. After the measurement of protein concentration by BCA kit (Beyotime), an aliquot (100 µg) of each extract was transferred to a 10 kD centrifugal filter (Merck Millipore), and 200 µL of 0.1 M triethylammonium bicarbonate (TEAB, Thermo Scientific) buffer was added to wash off lysis buffer twice at 14,000 g for 30 min at 4°C. Thereafter, 50 µL of 0.5 M TEAB was used to re-dissolve the protein residues in the ultrafilter, and then the proteins were denatured and reduced by incubating in a buffer, containing 0.1% SDS, 5.3 mM TCEP, and 0.5 M TEAB for 1 h at 60°C, followed by alkylation of cysteinyl thiols with 3 µL of 200 mM MMTS at 25°C for 15 min. The excess of denaturing, reducing, and alkylation reagents were washed off by centrifuging twice at 14,000 g for 30 min at 4°C with 200 µL of 0.1 M TEAB buffer, and the protein residue was re-dissolved with 30 µL of 0.5 M TEAB and digested by adding 2 µL of 1 μg/μL MS grade rAc-Trypsin (Bei-Er-Li, China) and incubating at 37°C for 16 h. The resulting tryptic peptides were collected by adding 200 µL 0.1 M TEAB buffer into the 10-kD centrifugal filter, spinning at 14,000 g for 30 min at 4°C, followed by desalting by a C18 cartridge (Waters Sep-Pak). The C18 cartridge was first activated by 1 ml of 0.1% formic acid (Honeywell Fluka™)/80% acetonitrile (Fisher Chemical) and 1 ml of 0.1% formic acid (FA)/50% acetonitrile (ACN) sequentially, and then equilibrated by 1 ml of 0.1% trifluoroacetic acid (Alfa Aesar) twice. The collected peptides were loaded into the equilibrated column and washed twice with 1 ml of 0.1% trifluoroacetic acid (TFA), and then eluted with 1 ml of 0.1% FA/50% ACN and 1 ml of 0.1% FA 80% ACN sequentially. Finally, the desalted peptides were merged and dried in a vacuum centrifuge for further use.

#### iTRAQ labeling

Each desalted peptide mixture was re-dissolved in 20 μL of 0.5 M TEAB buffer, and mixed with 70 μL of iTRAQ labeling reagent (SCIEX) dissolved in isopropanol (Cairn Pharmaceutical Technology Co.). The resulting mixture was adjusted to pH = 7.6 and then incubated at room temperature for 2 h. The labeling reaction was then quenched by adding 150 μL of deionized water and incubating for 15 min at room temperature. Thereafter, each labeled peptide mixture with different isotopic patterns from six groups were equivalently mixed and desalted by a C18 cartridge again following the same procedure described earlier.

#### HPLC pre-fractionation

The labeled and desalted peptide mixture was dried by vacuum centrifuge, and then re-dissolved in 100 uL 4.5 mM ammonium formate in 2% ACN aqueous solution (pH 10). An aliquot (95 uL) of each labeled peptide mixture was loaded into HPLC (Agilent Technologies 1260 infinity) with a C18 column (CAPCELL PAK MG II S-5, 4.6 × 150 mm, 5 μm, Shiseido), and eluted by basic (pH = 10) mobile phases A (4.5 mM HCOONH4 in 2% ACN) and B (4.5 mM HCOONH4 in 90% ACN). The gradient started with 0% B until 9 min and continuously increased to 6% B at 13 min, 28.5% B at 63 min, 34% B at 68.5 min, 60% B at 81.5 min, and maintained until 90 min with a flow rate of 1 ml/min. The fractions were collected chronologically into 12 tubes from 3 to 90 min, individually lyophilized and re-dissolved in 10 µL of 0.1% formic acid for NanoLC–MS/MS analysis.

#### NanoLC–MS/MS analysis

The NanoLC–MS/MS analysis was performed on an Orbitrap Fusion Lumos mass spectrometer (Thermo Fisher Scientific) coupled with an EASY-nLC 1200 nanoHPLC system equipped with an Acclaim™ PepMap™ 100 pre-column (20 mm × 75 μm, 3 µm) and an Acclaim™ PepMap™ RSLC C18 analytical column (150 mm × 75 μm, 2 µm). The mobile phases for the NanoLC separation were 0.1% FA in H2O (A) and 80% ACN/20% H2O (B), respectively, with a flow rate of 300 nL/min. The mobile phase B started at 2% and increased to 6% at 1 min, and then to 30% at 85 min, 60% at 94 min, and sharply to 90% within 1 min and remained at 90% for 5 min, and then decreased to 50% within 1 min and remained at 50% for 9 min, finally dropped down to 2% within 1 min and remained 3 min. An aliquot (1 μL) of each HPLC fraction containing 0.1% formic acid was loaded, and elution from the analytical column was directly infused into the mass spectrometer for MS/MS analysis. The details of the parameters of the MS/MS analysis are listed in Supplementary Table S1.

#### Protein identification and quantification

Acquired MS/MS spectra were analyzed by Proteome Discoverer 2.2 (Thermo Fisher Scientific) to identify and quantify proteins based on the protein database (Version 10/25/2017, 42,252 sequences) downloaded from the UniPort database. The taxonomy was *Rattus norvegicus*, and the taxon identifier was 10116. Sequest HT 2.2 search engine was used for peptide matching. The dynamic modifications were oxidation at methionine and phosphorylation at serine and threonine. The static modifications were carbamidomethylation at cystine, iTRAQ labeling at lysine, tyrosine, and N-terminus of all peptides. The quantitative results were normalized based on the abundance of total peptides in each set. Abundance ratios were calculated for each protein identified in all groups other than controls by comparing the value of each protein to that of the same protein in the control group. The other settings are the defaults of PD software.

### Western blotting

The expression levels of the NPPA, TPM1, and TPM2 in the control and model groups were measured by Western blotting with glyceraldehyde phosphate dehydrogenase (GAPDH) as an internal control protein. An aliquot (40 µg) of protein extract from the heart tissue of each group was boiled at 95°C for 5 min in SDS-loading buffer and separated over 4–12% polyacrylamide gel (GeneScript) at 140 V for 1 h with Tris-MOPS-SDS running buffer (GeneScript). The proteins were then transferred to the PVDF membrane by semi-dry transfer blotting at 20 V for 1 h. The PVDF membrane (Millipore) with proteins was cut into two parts along ca. 30 kD, and then blocked in 5% milk TBST buffer for 120 min. Anti-NPPA antibody (Abcam), anti-TPM2 (Abcam), anti-TPM1 (Abcam), and anti-GAPDH (Abcam) were diluted at 1:1000, 1:300, 1:1000, and 1:5000, respectively, to blot PVDF membrane by incubation at 4°C for 10 h, and then washed three times by 5% milk TBST for 5 min each. The PVDF membrane was then incubated with horseradish peroxidase-conjugated second antibodies, goat antirabbit antibody (Abcam) for NPPA, TPM2, and TPM1 and goat antimouse antibody (Abcam) for GAPDH, respectively, for 1 h at 25°C. After washing with TBST, the protein bands were visualized by enhanced chemiluminescence (Tanon 5200Multi, China), and the optical densities were quantified using ImageJ.

### Bioinformatics analysis

The proteins identified and quantified by MS/MS, of which false-discovery rates (FDRs) are <0.01, *p*-values <0.05, and abundance ratios (model vs. control) ≥ 1.5 or ≤0.67, were uploaded into the STRING 10.0 data pool for Gene Ontology (GO) and protein–protein interaction (PPI) network analysis. The UniPort accession ID of the differentially expressed proteins (DEPs) in the heart tissues of the model and treated groups were inputted into the list of gene name box, and *Rattus norvegicus* organism was selected for the analysis. The confidence option was selected for the meaning of network edge, and textmining, experiments, databases, co-expression, neighborhood, gene fusion, and co-occurrence options were chosen for active interaction sources.

### Statistical analysis

All data were analyzed with Origin 8.0 and Microsoft Office Excel 2010 software and are expressed as mean ± SEM. The between-group differences in physiological and/or pathological indexes were analyzed by one-way ANOVA. A two-sided *p* value <0.05 represents statistically significant. * indicates *p* < 0.05, **p < 0.01, and ***p < 0.001. Principal component analysis (PCA) was performed on MATLAB(Version 8.4.0.150421, MathWorks Inc.).

## Results

### Characterization of components in *Descurainia sophia* seeds and *Astragalus mongholicus*

**FIGURE 2:**
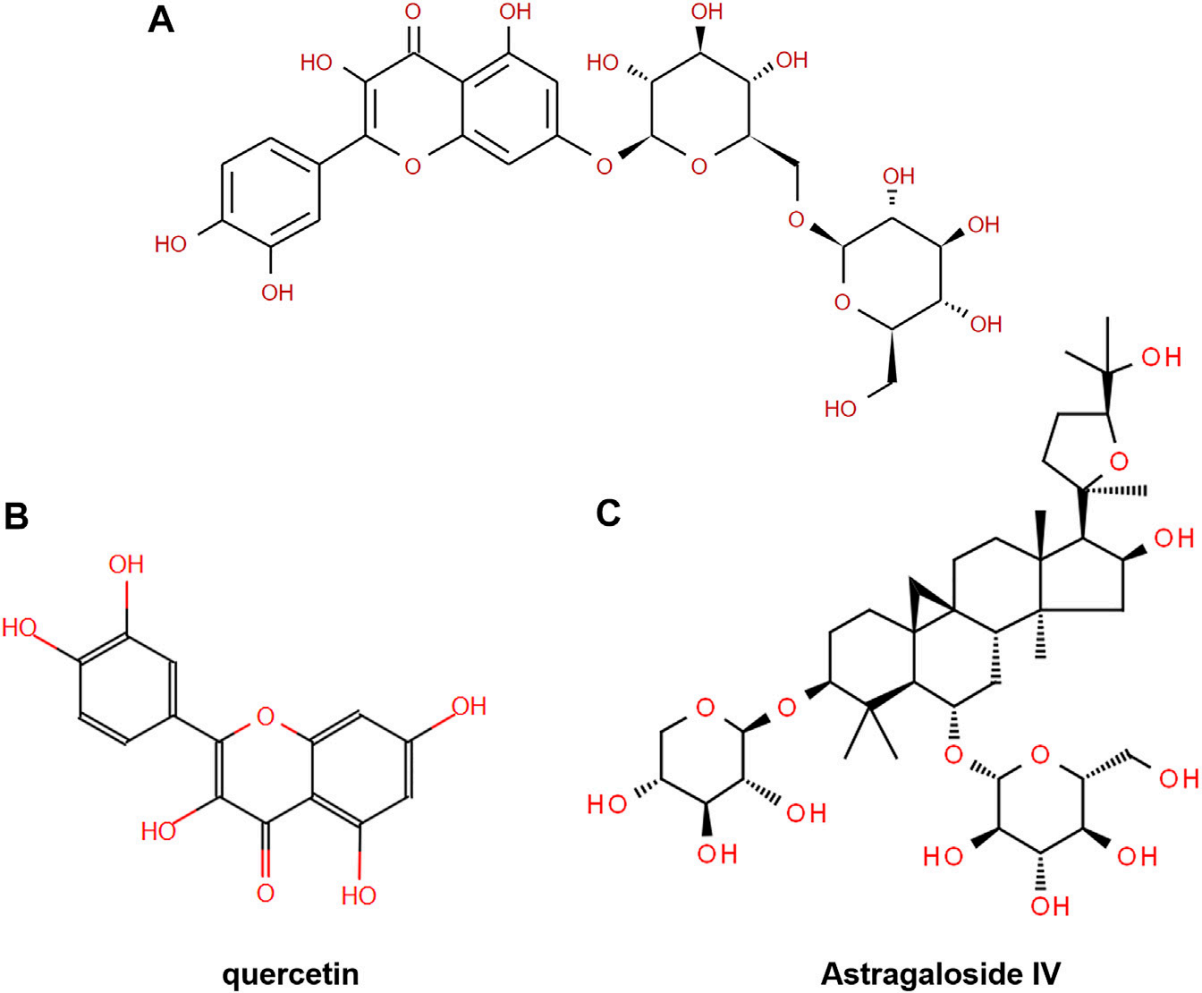
Chemical structure of **(A)** quercetin-7-O-β-D-glucopyranosyl (1→6)-β-D-glucopyranoside, **(B)** quercetin, and **(C)** astragaloside IV.

To investigate the mechanism of action of *Descurainia sophia* seeds (DS) and *Astragalus mongholicus* (AM), we first characterize the main components in DS and AM decoctions, individually, by UPLC–MS/MS. As shown in Supplementary Figure S1, both the decoctions display complicated composition. We collected all ion peaks detected by MS in DS and AM decoctions under both positive and negative modes into the Progenesis QI program for principal component analysis (PCA). In total, 10,078 positive ion peaks and 6,710 negative ion peaks were selected by Progenesis QI. The results showed that under the positive mode, the first principal component (PC1) can distinguish DS and AM, and under the negative mode, the second principal component (PC2) can separate DS and AM (Supplementary Figure S2). The 10,078 positive ion peaks and 6,710 negative ion peaks enabled the identification of 90 compounds commonly existing in DS and AM decoctions, of which the abundance (A) ratios calculated on A_DS_/A_AM_ or A_AM_/A_DS_ are >1 and <25 (Supplementary Figure S2). Furthermore, after data filtering, 48 and 23 compounds were exclusively identified for DS with the A_DS_/A_AM_ values > 25, and for AM with the A_AM_/A_DS_ >25, respectively (Tables 1, 2, Supplementary Tables S3, S4). The identification was verified further by checking the MS^2^ fragment patterns and the isotope similarity of each compound to the standard. For example, one of the main components in DS is quercetin-7-O-β-D-glucopyranosyl (1→6)-β-D-glucopyranoside (Figure 2A), according to the integrative pharmacology-based network computational research platform of traditional Chinese medicine database (TCMIP v2.0, http://www.tcmip.cn/). The hydrolytic product of this component, quercetin (Figure 2B), was identified under positive mode with a retention time of 7.66 min at the extraction chromatogram (EIC) (DS-11 in Supplementary Figure S3A), and the fragment pattern is depicted in Supplementary Figure S4A, which shows that the MS/MS fragments of quercetin were observed at *m/z* 153.0183, 121.0284 and 137.0234, being consistent with Makarov’s report (Scigelova et al., 2011). The well-known major component in AM, astragaloside IV (Figure 2C), was detected at 13.13 min (AM-23 in Supplementary Figure S3C), of which the MS/MS fragment ions were detected at *m/z* 669.3967, 627.3853, and 203.0526 (Supplementary Figure S4B).

**TABLE 1:** Components identified in *Descurainia sophia* seed decoction.

Compound no.	Accepted description	Retention time/min	m/z	Adducts	Formula	Abundance	Fold change^a^
DS-1	(5Z)-3-Ethyl-5-{4-[(4-fluorobenzyl)oxy]benzylidene}-2-thioxo-1,3-thiazolidin-4-one	3.82	372.0534	M-H	C_19_H_16_FNO_2_S_2_	2306842	120.8
DS-2	1-[(Benzyloxy)carbonyl]-3-(carboxymethyl)-3-pyrrolidinecarboxylic acid	7.04	308.1125	M + H	C_15_H_17_NO_6_	1018277	31.0
DS-3	1-Nitro-7-hydroxy-8-glutathionyl-7,8-dihydronaphthalene	10.30	477.1080	M-H_2_O-H	C_20_H_24_N_4_O_9_S	963011	162.1
DS-4	(8Z,22Z)-4-Methoxy-2-oxa-11,16,20-triazatricyclo [22.2.2.1–3.7∼]nonacosa-1(26),3(29),4,6,8,22,24,27-octaene-10,21-dione	5.68	472.2168	M + Na	C_26_H_31_N_3_O_4_	875325	2199.6
DS-5	Methyl 5-({(4-carbamoyl-2,6-dimethyl-N-{[(2-methyl-2-propanyl)oxy]carbonyl}-L-phenylalanyl)[(1S)-1-(5-phenyl-1H-imidazol-2-yl)ethyl]amino}methyl)-2-methoxybenzoate	8.94	718.3035	M + Cl	C_38_H_45_N_5_O_7_	841332	2231.0
DS-6	1-(1-Ethyl-1H-pyrrol-3-yl)-7,7-difluoro-2-azaspiro [3.5]nonane	9.87	289.1266	M + Cl	C_14_H_20_F_2_N_2_	831149	1688.2
DS-7	Nepetin	7.79	317.0653	M + H	C_16_H_12_O_7_	803804	268.4
DS-8	Isorhamnetin	8.27	317.0654	M + H	C_16_H_12_O_7_	722166	160.2
DS-9	(−)-Epothilone A	9.33	532.2177	M + K	C_26_H_39_NO_6_S	671218	33.8
DS-10	4,4-Difluoro-2-({[(2-methyl-2-propanyl)oxy]carbonyl}amino)cyclopentanecarboxylic acid	7.71	264.1039	M-H	C_11_H_17_F_2_NO_4_	561303	6441.1
DS-11	Quercetin	7.66	303.0493	M + H	C_15_H_10_O_7_	503905	203.8
DS-12	(8E,22E)-4-Methoxy-2-oxa-11,16,20-triazatricyclo [22.2.2.1–3.7∼]nonacosa-1(26),3(29),4,6,8,22,24,27-octaene-10,21-dione	5.98	472.2164	M + Na	C_26_H_31_N_3_O_4_	382457	35.9
DS-13	Lawsone	6.91	175.0377	M + H	C_10_H_6_O_3_	327344	25.0
DS-14	Ethyl 3-amino-5-methoxy-4.5,6,7-tetrahydro-1-benzofuran-2-carboxylate	7.04	262.1069	M + Na	C_12_H_17_NO_4_	323375	33.0
DS-15	Kaempferol	10.31	287.0550	M + H	C_15_H_10_O_6_	315159	25.8
DS-16	4(1H)-Isoquinolinone	6.16	146.0589	M + H	C_9_H_7_NO	308144	58.1
DS-17	DSS	9.56	777.2218	M + Na	C_34_H_42_O_19_	272640	280.6
DS-18	Tozadenant	8.45	429.1548	M + Na	C_19_H_26_N_4_O_4_S	261991	126.7
DS-19	Hypolaetin	7.09	303.0491	M + H	C_15_H_10_O_7_	247943	157.2
DS-20	1,2-indandione	6.96	147.0431	M + H	C_9_H_6_O_2_	212572	29.4
DS-21	1-O-trans-cinnamoyl-Î^2^-D-glucopyranose	5.85	291.0898	M-H_2_O-H	C_15_H_18_O_7_	205788	3514.0
DS-22	SAMe	8.63	381.1330	M + H-H_2_O	C_15_H_22_N_6_O_5_S	196306	29.1
DS-23	(3-Isopropoxy-2-thienyl) (phenyl)methanol	6.96	247.0778	M-H	C_14_H_16_O_2_S	192040	257871.9
DS-24	Methyl (2Z)-4-(2,3-dihydro-1,4-benzodioxin-6-yl)-2-hydroxy-4-oxo-2-butenoate	8.20	287.0505	M + Na	C_13_H_12_O_6_	187762	43.9
DS-25	(2S,3Z)-5-{[(2R,3R,5S,6S)-6-{(2E,4E)-5-[(3R,4R,5R)-4-Hydroxy-7,7-dimethyl-1,6-dioxaspiro [2.5]oct-5-yl]-3-methyl-2,4-pentadien-1-yl}-2,5-dimethyltetrahydro-2H-pyran-3-yl]amino}-5-oxo-3-penten-2-yl acetate	4.39	506.3137	M + H	C_28_H_43_NO_7_	157104	419.8
DS-26	(4-Methoxy-3,5-dimethyl-2-thienyl) (phenyl)methanol	8.63	247.0780	M-H	C_14_H_16_O_2_S	148151	2318.7
DS-27	(2E)-N-(6-Amino-1,3-diethyl-2,4-dioxo-1.2,3,4-tetrahydro-5-pyrimidinyl)-3-(3,4-dimethoxyphenyl)acrylamide	8.89	427.1389	M + K	C_19_H_24_N_4_O_5_	122759	85.2
DS-28	1-[(4-Methoxyphenyl)sulfonyl]-3-(4.4,5,5-tetramethyl-1,3-dioxolan-2-yl)-1H-pyrrolo [2,3-b]pyridine	9.87	451.1124	M + Cl	C_21_H_24_N_2_O_5_S	115812	Infinity
DS-29	Hexamethylolmelamine	9.74	287.1109	M-H_2_O-H	C_9_H_18_N_6_O_6_	94535	1208.7
DS-30	2-Methyl-2-propanyl [5-(6-hydroxy-2-naphthyl)-2,2-dimethyl-1,3-dioxan-5-yl]carbamate	7.71	354.1675	M-H_2_O-H	C_21_H_27_NO_5_	92513	Infinity
DS-31	Convalloside	11.28	735.3238	M + Na	C_35_H_52_O_15_	81129	28.1
DS-32	Mirificin	6.86	547.1655	M-H	C_26_H_28_O_13_	78320	2798.7
DS-33	Bis [(3R,4S,5S,6R)-3,4,5-trihydroxy-6-(hydroxymethyl)tetrahydro-2H-pyran-2-yl] malonate	7.48	427.1103	M-H	C_15_H_24_O_14_	70481	1752.4
DS-34	4-Hydroxy-3-methyl-6-(trifluoromethyl)-4.5,6,7-tetrahydro-1-benzofuran-2-carboxylic acid	7.66	287.0493	M + Na	C_11_H_11_F_3_O_4_	68140	144.5
DS-35	N-Hydroxy-2-(4-phenyl-1,3-thiazol-2-yl)ethanimidamide	8.63	232.0549	M-H	C_11_H_11_N_3_OS	66872	2358.5
DS-36	(6E)-4-Hydroxy-1,7-diphenyl-6-hepten-3-one	9.95	303.1333	M + Na	C_19_H_20_O_2_	59840	43.1
DS-37	5-[2-(Ethylsulfanyl)phenyl]-3-methyl-5-oxopentanoic acid	8.58	265.0878	M-H	C_14_H_18_O_3_S	48253	12299.5
DS-38	(3Z)-2,10-Diamino-4-(phosphonomethyl)-3-decenoic acid	8.63	329.1039	M + Cl	C_11_H_23_N_2_O_5_P	46056	214.8
DS-39	1,2-O-Cyclohexylidene-a-D-glucofuranose	8.58	295.0971	M + Cl	C_12_H_20_O_6_	46002	Infinity
DS-40	Benzyl 5-[(acetylsulfanyl)methyl]-4-(1,3-benzodioxol-5-yl)-1-hydroxy-l-prolylglycinate	6.65	467.1278	M-H_2_O-H	C_24_H_26_N_2_O_7_S	42389	2324.0
DS-41	GDC-0152	6.60	479.2189	M-H_2_O-H	C_25_H_34_N_6_O_3_S	42040	172.2
DS-42	Sakuranin	10.74	471.1286	M + Na	C_22_H_24_O_10_	36290	38.4
DS-43	Aklaviketone	8.58	411.1090	M + H	C_22_H_18_O_8_	19893	25.9
DS-44	1-[4-({(1R)-1-[(6S,7S)-2-Amino-7-methyl-4-oxo-1,4,5,6,7,8-hexahydro-6-pteridinyl]ethyl}amino)phenyl]-1-deoxy-5-O-(5-O-{[(1S)-1,3-dicarboxypropoxy](hydroxy)phosphoryl}-alpha-D-ribofuranosyl)-D-ribitol	8.81	799.2547	M + Na	C_30_H_45_N_6_O_16_P	11051	33.2
DS-45	Neohesperidin dihydrochalcone	8.45	635.1893	M + Na	C_28_H_36_O_15_	7239	42.8
DS-46	N-[(9H-Fluoren-9-ylmethoxy)carbonyl]-N-methyl-1-{[(2-methyl-2-propanyl)oxy]carbonyl}histidine	9.56	530.1665	M + K	C_27_H_29_N_3_O_6_	6166	51.8

a Fold change = (abundance)DS/(abundance)AM

**TABLE 2:** Components identified in *Astragalus mongholicus* decoction.

Compound No.	Accepted description	Retention time/min	m/z	Adducts	Formula	Abundance	Fold change^a^
AM-1	Deslanoside	11.90	941.4749	M-H	C_47_H_74_O_19_	987131	270.0
AM-2	2′-Methyl-1H,1′H-2.5′-bibenzimidazole	9.20	283.0763	M + Cl	C_15_H_12_N_4_	973789	1673.7
AM-3	(11R,23R)-14,17,20-Trihydroxy-14,20-dioxido-8,26-dioxo-9,13,15,19,21,25-hexaoxa-14lambda∼5∼,20lambda∼5∼-diphosphatritriacontane-11,23-diyl dioctanoate	13.26	885.4538	M-H_2_O-H	C_41_H_78_O_17_P_2_	948470	385.7
AM-4	9,10-Dihydrobenzo [e]acephenanthrylene-5,9,10-triol	8.94	283.0764	M-H_2_O-H	C_20_H_14_O_3_	890204	304.5
AM-5	8-Azaadenosine	9.95	267.0823	M-H	C_9_H_12_N_6_O_4_	857889	458.3
AM-6	Decursinol	10.66	269.0800	M + Na	C_14_H_14_O_4_	829194	37.0
AM-7	Seocalcitol	13.08	437.3415	M + H-H_2_O	C_30_H_46_O_3_	723031	43.9
AM-8	(2E)-3-[2-(3-Ethylphenoxy)-5-fluorophenyl]acrylic acid	11.67	267.0823	M-H_2_O-H	C_17_H_15_FO_3_	690119	43.6
AM-9	8′-apo-beta-carotenol	13.08	419.3309	M + H	C_30_H_42_O	630918	94.6
AM-10	2-Ammonio-5-[(1-carboxy-2-{[(1Z)-N-hydroxy-2-phenylethanimidoyl]sulfanyl}ethyl)amino]-5-oxopentanoate	2.64	382.1102	M-H	C_16_H_21_N_3_O_6_S	594536	142.8
AM-11	2-Phenyl-7-quinolinecarbaldehyde	8.94	268.0536	M + Cl	C_16_H_11_NO	541619	Infinity
AM-12	OLMESARTAN LACTONE	10.61	463.1659	M + Cl	C_24_H_24_N_6_O_2_	430921	123.8
AM-13	(6S,9S,9aS)-N-Benzyl-6-(4-hydroxybenzyl)-2,9-dimethyl-4,7-dioxo-8-(8-quinolinylmethyl)hexahydro-2H-pyrazino [2,1-c][1,2,4]triazine-1(6H)-carboxamide	9.02	577.2606	M-H	C_33_H_34_N_6_O_4_	306730	244.4
AM-14	3-Hydroxylup-18-en-21-one	12.77	423.3613	M + H-H_2_O	C_30_H_48_O_2_	292174	55.0
AM-15	Dicyclohexyl [2-(2,6-dimethoxybenzyl)benzyl]phosphine	7.40	461.2595	M + Na	C_28_H_39_O_2_P	110198	52.7
AM-16	Ganoderal A	12.11	437.3410	M + H	C_30_H_44_O_2_	106769	107.5
AM-17	Astragaloside III	12.95	807.4493	M + Na	C_41_H_68_O_14_	95644	30.2
AM-18	Astragaloside II	13.08	849.4615	M + Na	C_43_H_70_O_15_	92457	43.1
AM-19	Metoprolol succinate	13.83	653.4049	M + H	C_34_H_56_N_2_O_10_	83919	1307.8
AM-20	Soyasaponin I	12.77	965.5083	M + Na	C_48_H_78_O_18_	75689	152.5
AM-21	N∼2∼-[(8-Fluoro-6-{5-[(2-methyl-2-propanyl)sulfamoyl]-3-pyridinyl}[1,2,4]triazolo [1,5-a]pyridin-2-yl)carbamoyl]-N,N-dimethylglycinamide	2.02	473.1553	M-H_2_O-H	C_20_H_25_FN_8_O_4_S	75544	28.4
AM-22	(5R)-2,4-Dideoxy-1-C-{(2S,3R,4S)-3-hydroxy-4-[(2R,3S,4E,6E,9R,10S,11R,12E,14Z)-10-hydroxy-3,15-dimethoxy-7.9,11,13-tetramethyl-16-oxooxacyclohexadeca-4.6,12,14-tetraen-2-yl]-2-pentanyl}-5-isopropyl-4-methyl-alpha-D-threo-pentopyranose	13.26	645.3974	M + Na	C_35_H_58_O_9_	49716	70.1
AM-23	Astragaloside IV	13.13	807.4506	M + Na	C_41_H_68_O_14_	42693	34.4

a ^Fold change = (abundance)AM/(abundance)DS^

### Phenotypic and pathological characterization of isoproterenol-induced cardiomyopathy in rats

Isoproterenol (ISO) as a β-adrenergic receptor (β-AR) agonist is often used to induce cardiomyopathy in the mouse (Shanmugam et al., 2019; Houson et al., 2020; Zhu et al., 2020). We have previously constructed rat models with the upper energizer stage (ICD code: SG70) and fluid retention patterns (ICD code: SF11) by injecting subcutaneously into the abdomen of rats, accompanied by the endotracheal intubation. The established models included disorders in both the heart and lung of rats (Xie et al., 2015). We have also primarily evaluated the effects of DS, AM, and DS/AM formula on the lung and heart functions of the model rats and found that the combined use of DS with AM produced better pharmacological effects on the lung and cardiac functions evidenced by the reversion of the lung weight index (LWI), the lung permeability index (LPI), left ventricular weight index (LVWI), and left ventricular ejection fraction (LVEF) (Chen et al., 2019). In the present work, we only evaluate the phenotypes and pharmacological benefits of DS and AM as well as each combination on the disorders in the heart of the rat models induced by isoproterenol.

**FIGURE 3:**
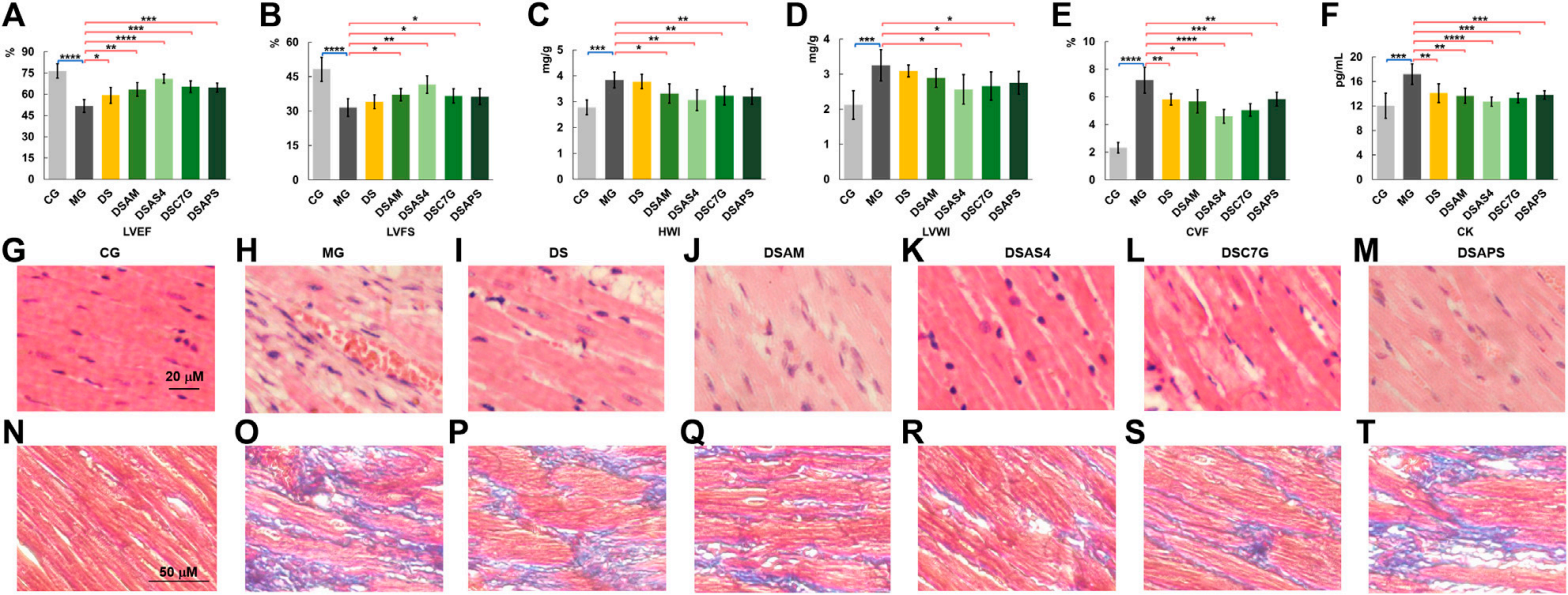
Phenotypic and pathological characterization **(A–F),** left ventricular ejection fraction (LVEF, **(A)**, left ventricular fraction shortening (LVFS, **(B)**, heart index (HWI, **(C)**, left ventricular mass index (LVWI, **(D)**, collagen volume fraction (CVF, **(E)** of myocardial cells, and plasma CK levels **(F)** of rats of the control (CG), model (MG), and treated groups with *Descurainia sophia* seed decoction alone (DS), and *Descurainia sophia* seeds plus *Astragalus mongholicus* decoction (DSAM), *Descurainia sophia* seeds decoction plus astragaloside IV (DSAS4), calycosin-7-glucoside (DSC7G), or Astragalus polysaccharides (DSAPS). The results of LVEF, LVFS, HWI, LVWI, CVF, and CK were measured by randomly selecting six rats (n = 6) from each group and are represented as mean ± SD. *p < 0.05 and **p < 0.01, ***p < 0.001 and ****p < 0.0001, represent statistical significance and high significance, respectively. **(G–M)** H&E (400×) and **(N–T)** Masson’s (200×) trichrome staining of heart tissues of all groups. For Masson’s staining, collagen fibers were stained in blue, and myocardial cell in dark red.

As echocardiographic left ventricular ejection fraction (LVEF) and left ventricular fraction shortening (LVFS) accurately reflect the cardiac function, for example, systolic and diastolic functions (Cho et al., 2018; Rieth et al., 2019), we first performed a high-frequency echocardiographic examination on rats in all groups. As shown in Supplementary Figure S5, significant changes were observed between the ventricular cavity size and wall motion coordination of the heart of rats in the control group and model group. Compared with the control group (CG), the average (n = 6) LVEF and LVES of the model group (MG) reduced from 76.4 to 51.6%, and 48.2 to 31.5%, respectively (Figures 3A,B). Although the changes in LVEF and LVES between the controls and models were not as significant as those observed in patients with newly diagnosed DCM and heart failure (Cho et al., 2018), the alterations in LVEF and LVES together with the changes in the cardiac index (HWI) and left ventricular mass index (LVWI) between the controls and the models (Figures 3C,D), demonstrated that the models had developed pseudo-cardiomyopathy, termed as isoproterenol-induced cardiomyopathy (ISO-iCM) (Zhu et al., 2020).

Next, we evaluate the pharmacological effects of *Descurainia sophia* seeds (DS) and the combination of DS with *Astragalus mongholicus* (AM), astragaloside IV (AS4), calycosin-7-glucoside (C7G), and Astragalus polysaccharides (APS) on ISO-iCM in rats, based on the effect of various treatments on LVEF, LVFS, LVWI, and HWI levels of models. We found that AM and C7G slightly improved the pharmacological efficacy of DS, and AS4 significantly promoted the pharmacological effect of DS, whereas PLS showed little benefit for the pharmacological efficiency of DS on ISO-iCM (Figures 3A–D).

To evaluate further pharmacological efficiency of various combinations of DS with AM and the three active components of AM, we performed histopathological assays on the left ventricular myocardial tissues. The H&E staining results (Figures 3G,H) indicated that compared to the controls, the myocardial cells in the heart of models were significantly enlarged and hypertrophic and disorderly arranged accompanied with increased intercellular substances. Moreover, the inflammatory cell infiltration in the heart of the model was obvious. After treatment with DS or the combinations, these pathological phenotypes were diminished to a different extent (Figure 3I-M). Notably, the combined uses of DS with AS4 or C7G again resulted in further reduction in the severity of hypertrophy of myocardial cells and the content of intercellular substances in the hearts, compared with that treated with DS alone (Figures 3I,K, L), implying better pharmacological benefits on the heart injury in the model rats. However, the combination of DS with APS did not produce an improvement in the pharmacological efficiency of DS to ISO-iCM in rats (Figure 3M).

We also performed Masson’s staining on the heart tissues of all groups (Figures 3N–P), where collagen fibers were stained in blue and myocardial cells in dark red. Based on Masson’s staining, the average cardiac collagen volume fractions (CVFs) of each group were calculated and depicted in Figure 3E. The results indicate that compared to the controls, the CVF value of model rats significantly increased (*p* < 0.01) but decreased to a large content after treatments, though the treatments did not reverse completely the CVF level of models. Again, we found that the treatment with DS combined with AS4 exhibited the highest effectiveness to reverse the CVF level (Figure 3R). Meanwhile, this combined treatment also reverses the plasma myocardial enzyme creatine kinase (CK) level of model rats to close to that of controls (Figure 3F). Given the increased expression of CK in plasma is an important diagnostic marker of myocardial ischemia (Holmvang et al., 1998), these results indicate that the treatment with DS and AS4 significantly alleviated myocardial cell necrosis, resulting from myocardial ischemia in rats with ISO-iCM.

### Identification of differential expressed proteins

**FIGURE 4:**
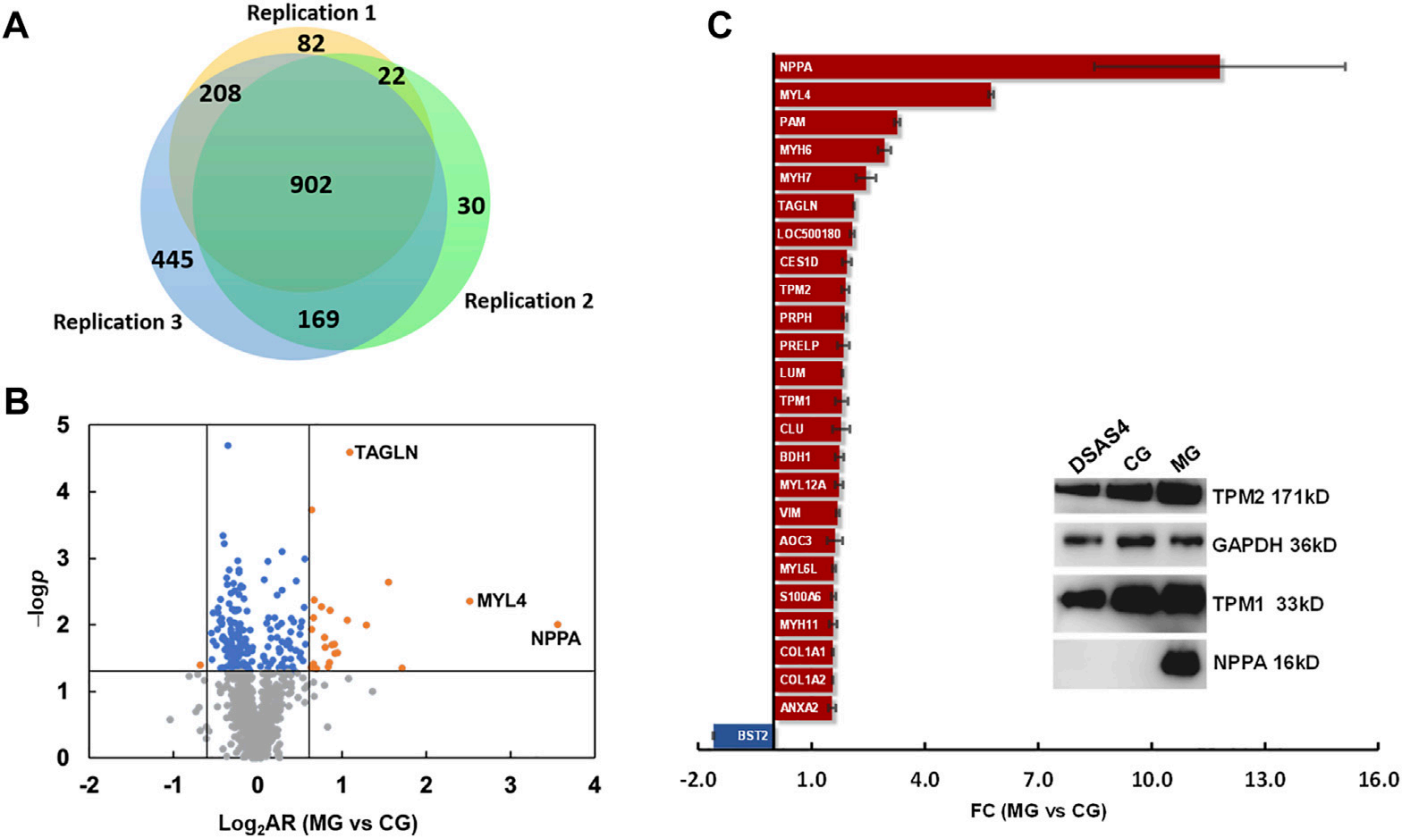
Differential expression proteomics of the heart of rats in the control group (CG) and ISO-iCM model group (MG). **(A)** Number of proteins identified in three parallel replicates. **(B)** Volcanic map of the proteins identified in both the control (CG) and model (MG) groups with various abundance ratios (ARs) and *p*-values. Orange point refers to a protein with a log_2_AR (MG vs. CG) of ≥0.59 or ≤ −0.59 and a *p*-value of <0.05; blue to a protein with a log_2_AR of > −0.59 or <0.59 and a *p*-value of <0.05, gray to a protein with a *p*-value of >0.05. **(C)** Fold changes (FCs) of 25 differentially expressed proteins in the heart of the model group (MG) compared to those in the control group (CG). The insert is the Western blotting bands of the representatives of DEPs, NPPA, TMP1, and TMP2 in the heart of rats in CG, MG, and the treated group with *Descurainia sophia* seeds decoction plus astragaloside IV (DSAS4). GAPDH was used as an internal reference.

After phenotypic characterization and physiological evaluation of rats with ISO-iCM before and after various treatments, we performed mass spectrometry (MS)-based quantitative proteomics analysis on the heart of rats to investigate the pharmacological efficiency of various treatments at a molecular level. The protein extraction from heart tissues and the subsequent MS/MS analysis were carried out in three independent replicates. In total, peptide matching spectrum maps allowed us to identify 55,307 peptides and 10,418 peptide groups, corresponding to 1,858 proteins. Of these 902 proteins were detected in three parallel sets, and 213 proteins were considered statistically reliable with a *p*-value <0.05 (Figures 4A,B, Supplementary Table S5). Among the 213 proteins were 24 proteins upregulated by more than 1.5-fold and 1 protein downregulated by more than 1.5-fold in the model rats compared to the counterparts in the controls (Figure 4C).

Among the 25 differentially expressed proteins (DEPs) (Figure 4C), the most significantly upregulated protein in the rats with ISO-iCM was natriuretic peptides A (NPPA), of which the fold-change (FC), designated as the abundance ratio of an upregulated protein in MG vs CG, but as negative reciprocal of abundance ratio of a downregulated proteins in MG vs CG, was 11.8. All 25 DEPs were grouped by the Proteome Discoverer (PD) software into extracellular matrix proteins, myosin family, intermediated filament family, actin or actin filament binding proteins, metabolism-related proteins, immune-related proteins, and calcium sensors (Table 3). It is worth to point out that the members of the myosin family, intermediated filaments, and actin filament binding proteins are all related to sarcomere modulation of the heart (Anderson et al., 2018; Daniels et al., 2021; Lehman et al., 2022). More importantly, the myosin proteins MYL4, MYH6, and MYH7, which work with actin to regulate the myocardial contraction (Daniels et al., 2021), were remarkably upregulated in the heart of the rats with ISO-iCM. This with the most significant upregulation of the commonly accepted biomarker NPPA to cardiomyopathy confirms the successful establishment of the ISO-iCM models at the molecular level.

**TABLE 3:** Differentially expressed proteins in the heart of rats with ISO-iCM.

Category	Accession	Gene symbol	Protein name (abbreviation)
Extracellular matrix protein	O08590	*Aoc3*	Membrane primary amine oxidase (AOC3)
	Q07936-1	*Anxa2*	Annexin A2 (ANXA2)
	P02466	*Col1a2*	Collagen alpha-2(I) chain (COL1A2)
	P02454	*Col1a1*	Collagen alpha-1(I) chain (COL1A1)
	P05371	*Clu*	Clusterin (CLU)
	P51886	*Lum*	Lumican (LUM)
	Q9EQP5	*Prelp*	Prolargin (PRELP)
	P14925-1	*Pam*	Peptidyl-glycine alpha-amidating monooxygenase (PAM)
Myosin family protein	Q63862-1	*Myh11*	Myosin-11 (MYH11)
	P13832	*Myl12a*	Myosin regulatory light chain RLC-A (MYL12A)
	Q64119-2	*Myl6l*	Myosin light polypeptide 6 (MYL6L)
	P02564	*Myh7*	Myosin-7 (MYH7)
	P02563	*Myh6*	Myosin-6 (MYH6)
	P17209	*Myl4*	Myosin light chain 4 (MYL4)
Intermediated filaments	P31000	*Vim*	Vimentin (VIM)
	P21807	*Prph*	Peripherin (PRPH)
Actin filament binding protein	P31232	*Tagln*	Transgelin (TAGLN)
	P04692-2	*Tpm1*	Isoform 2 of tropomyosin alpha-1 chain (TPM1)
	P58775-2	*Tpm2*	Isoform 2 of tropomyosin beta chain (TPM2)
Metabolism-related protein	P29147	*Bdh1*	D-beta-hydroxybutyrate dehydrogenase (BDH1)
	P16303	*Ces1d*	Carboxylesterase 1D (CES1D)
	P01161	*Nppa*	Natriuretic peptides A (NPPA)
Immunity-related protein	Q811A2	*Bst2*	Bone marrow stromal antigen 2 (BST2)
	P01835	Igkc	Ig kippa chain C (LOC500180)
Calcium sensor	P05964	*S100a6*	Protein S100-A6 (S100A6)

**FIGURE 5:**
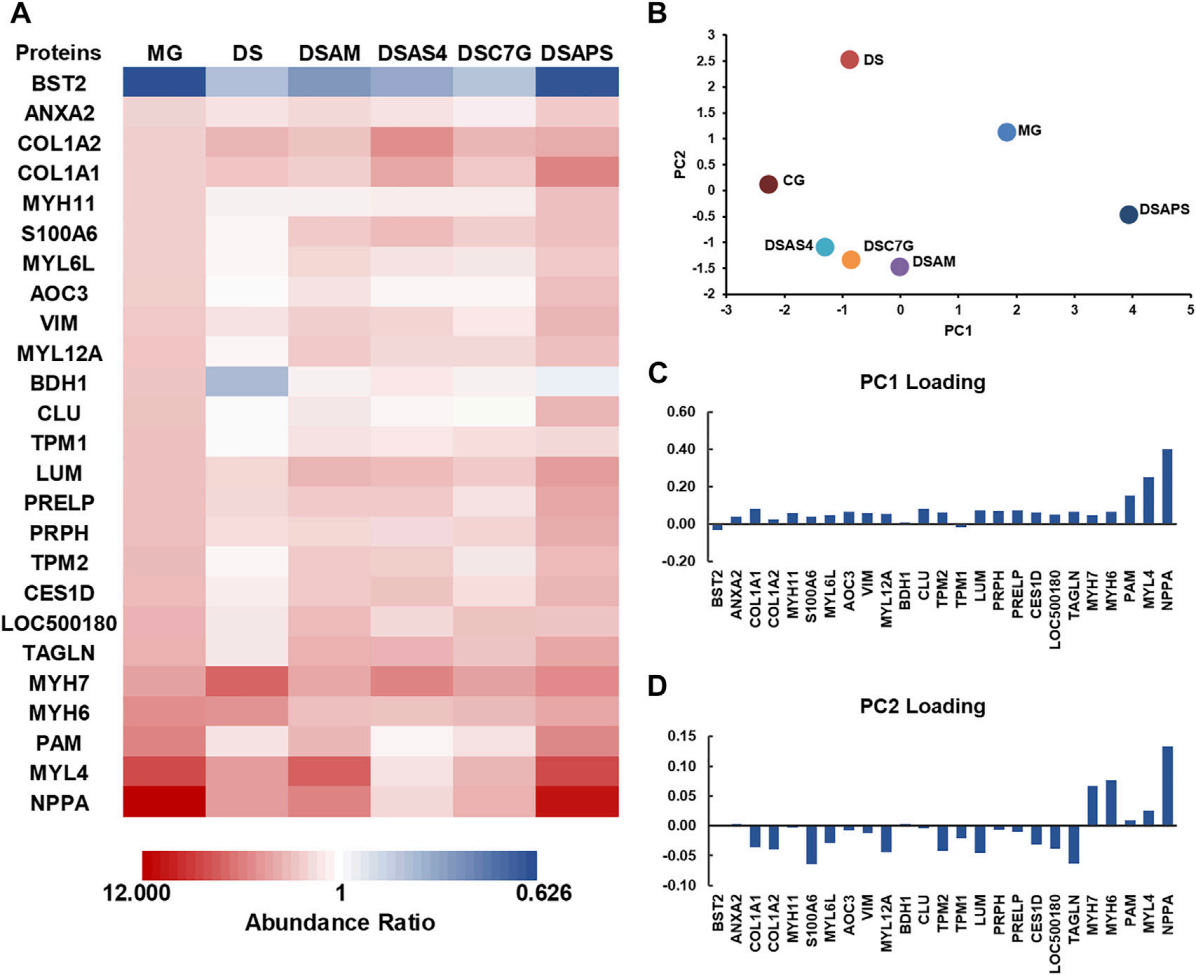
Differentially expressed proteins (DEPs) were detected in the heart of rats with ISO-iCM before and after treatments, compared to the controls. **(A)** Abundance ratios (model or treated groups vs. control group) of 25 DEPs in the model group (MG), treated group with Descurainia *sophia* seed decoction alone (DS), and *Descurainia sophia* seeds plus *Astragalus mongholicus* decoction (DSAM), *Descurainia sophia* seed decoction plus astragaloside IV (DSAS4), calycosin-7-glucoside (DSC7G), or astragalus polysaccharides (DSAPS). **(B)** Principal component analysis (PCA) is based on 213 proteins identified in all groups with a *p*-value <0.05. **(C,D)** Loading scores discriminate the model (MG) and treated groups, DS, DSAM, DSAS4, DSC7G, and DSAPS, over the control group (CG).

Next, we performed MS quantitative proteomics analysis on the heart of the five treated groups. As shown in Figure 5A and the Supplementary Table S5, subjected to the treatment with DS alone, the expression of the most 25 DEPs were reversed to a different extent except for COL1A2, COL1A1, BDH1, MYH7, and MYH6, among which COL1A1, COL1A2, MYH6, and MYH7, especially MYH7, were further regulated, but d-β-hydroxybutyrate dehydrogenase (BDH1) in mitochondrion was downregulated, compared to the models. In TCM practices, DS is often used in combination with AM to treat cardiac diseases (Yu et al., 2021). However, our proteomic data showed that this combination did not pronouncedly improve the pharmacological effectiveness, though AM appeared to reverse the expression of BDH1, which was downregulated by DS, to the control level (Figure 5A). This may be contributable to the upregulation of NPPA and MYL4 by AM, compared to the DS treated group (Figure 5A). Interestingly, the major active component of AM, AS4 not only reversed the further expression of NPPA and myosin such as MYH6 and MYL4 closer to the control level but also reversed the expression of MYH7 and BDH1, which were upregulated and downregulated, respectively, by DS (Figure 5A). We also noticed that APS significantly upregulated MYL4 and NPPA, which were the most and the second upregulated proteins in the heart of model rats with ISO-iCM, compared with the DS-treated group. These results indicate that the upregulation of NPPA and MYL4, resulting from AM may be attributed to its active component APS (Figure 5A).

To verify the expression of the DEPs identified by quantitative proteomics analysis, we selected three NPPA, TPM1, and TPM2, the molecular weights of which locate at the low, medium, and high levels, respectively, to perform the traditional Western blot assays. As shown in the inset of Figure 4C, the results confirmed that compared to the control group (CG), NPPA, TPM1, and TPM2 were all significantly upregulated, and the combination of DS with AS4 remarkably reversed the expression of the three proteins.

We performed principal component analysis (PCA) based on the protein profiling, including all 213 proteins detected in the heart of all six groups. The results indicated that among the five treated groups, the combination of DS with AS4 (DSAS4) made the treated group closer to the control group in the PC1 direction (Figure 5B), suggesting the highest pharmacological effect of this combination on ISO-iCM in rats. This is most likely contributable to the reversion of expression of NPPA, MYL4, and PAM, which contributes the most to PC loading in the positive direction (Figures 5A–C). In contrast, the treatment with DS and APS (DSAPS) made the treated group further away from the control one in the PC1 positive direction (Figure 5B) because this treatment further upregulated the expression of NPPA, MYL4, and PAM (Figure 5C). In other words, NPPA, MYL4, and PAM may serve as potential predictors and biomarkers for the diagnosis and prognosis of cardiomyopathy.

### Bioinformatics analysis

**FIGURE 6:**
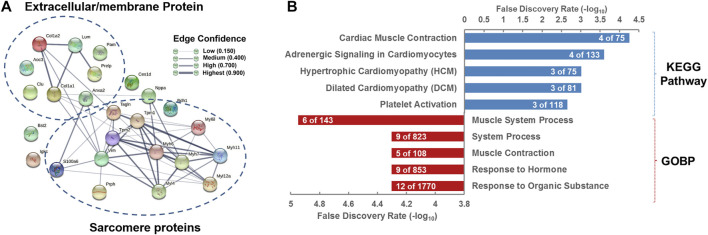
Bioinformatics analysis of differentially expressed proteins (DEPs) in the hearts of rats with ISO-iCM compared to the controls **(A)** Protein–protein interaction network of the 25 DEPs. **(B)** KEGG pathways and biological processes in which the 25 DEPs are associated with.

To further dissect the functions and roles of the 25 DEPs (Table 3) in the heart of rats with ISO-iCM, we next performed bioinformatics analysis on these proteins. First, the 25 DEPs were uploaded into STRING 11.0 for protein–protein interaction (PPI) analysis. As shown in Figure 6A, the PPI network of the 25 DEPs consists of 25 nodes (genes/proteins) and 54 edges (interactions), which indicates that 18 of the 25 DEPs interact with each other and that the interactions could be grouped at four levels based on the edge confidence scores. One interaction group with high confidence level covers four extracellular matrix (ECM) proteins, PRELP, LUM, COL1A1, and COL1A2, of which the primary function is cell connecting. Another interaction group with a high confidence level contains VIM, MYL4, MYH7, MYH11, MYH6, MYL12a, TAGLN, TPM2, and TPM1, which are all located in the cytoplasm and mainly regulate the structure and function of the sarcomere (Toepfer et al., 2020). Interestingly, being a member of type III intermediate filament proteins VIM directly interacts with the members of both the myosin family and ECM family. This suggests that VIM plays an essential role in ISO-iCM *via* involvement in the development of cytoskeleton and sarcomere, which are modulated by ECM proteins and myosin motor, respectively, associating with actin and/or actin-filament binding proteins such as TAGLN, TPM2, and TPM1.

**FIGURE 7:**
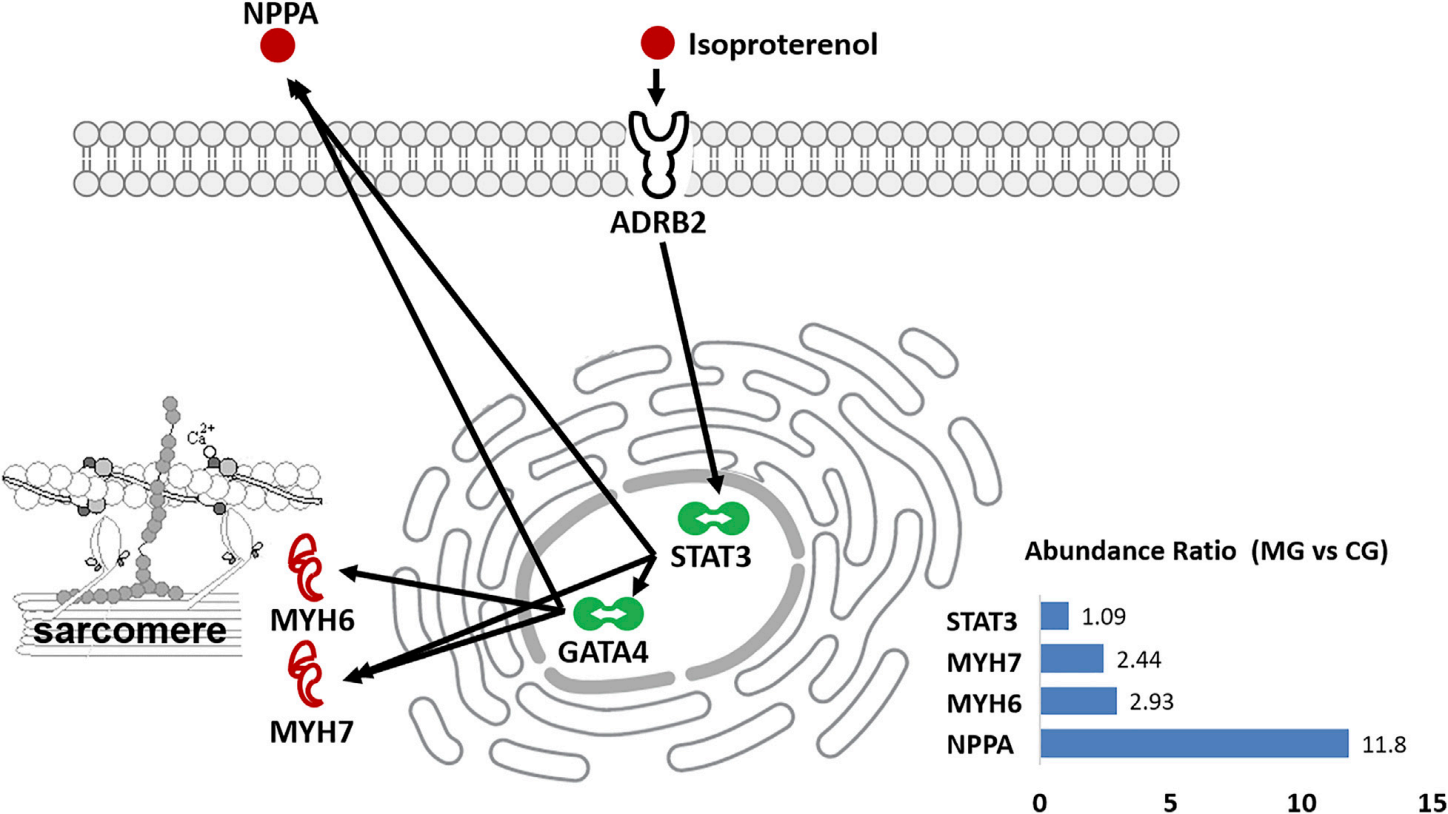
Diagrammatic process of isoproterenol-induced heart dysfunction in rats. It shows that isoproterenol binds to ADRB2 and upregulates the expression of NPPA, MYH6, and MYH7 *via* activating the transcriptional regulators STAT3 and GATA4. The inset shows the abundance ratio of the selected proteins expressed in the heart of controls (CG) and models (MG).

Natriuretic peptides A (NPPA) is an energy metabolism-related protein and mediates cardio-renal homeostasis by binding to and stimulating NPR1 to produce cGMP (Ogawa et al., 2004). As shown in Figure 6A, NPPA directly interacts not only with the myosin proteins such as MYH6 and MYH7, of which both are molecular motors of the sarcomere (Toepfer et al., 2020; Daniels et al., 2021) but also with actin cross-linking/gelling protein TAGLN (Huang et al., 2018b), actin filament binding proteins TPM1/2 (Rani et al., 2015), and the intermediate filament protein VIM (Hol and Capetanaki, 2017), which are all involved in the binding of myosin to actin, modulating the myosin motor (Anderson et al., 2018; Lehman et al., 2022). In addition, NPPA directly interacts with ECM protein COL1A1 weakly. These implicate that NPPA plays a crucial role in the development of ISO-iCM by participating regulation of sarcomere structure and function of cardiomyocytes. As mentioned earlier, we induced cardiomyopathy in rats by isoproterenol, which is a non-specific β-AR agonist and can increase inotropy, chronotropy, and systemic vasodilation (Verma et al., 2012). It is commonly accepted that isoproterenol binds to the β2 adrenergic receptor, ADRB2, of cardiomyocytes, which in turn regulates the function of the proteins such as SLC9A3R2, CD36, and STAT3 (Yin et al., 2003; Verma et al., 2012; Stapel et al., 2017). The activation of the signal transducer and activator of transcription 3 (STAT3) can rapidly upregulate the expression of some cellular proteins, including NPPA and MYH7 (Figure 7), which were significantly upregulated in rats with ISO-iCM (*vide supra*) and in the patients with DCM (Chan et al., 2020; Toepfer et al., 2020). On the other hand, STAT3 activates the transcription regulator GATA4 (Snyder et al., 2010; Wang et al., 2018), thereby upregulating the expression of NPPA, MYH7, and MYH6 (Figure 7), which interacted with each other to regulate the structure and function of the sarcomere, resulting in heart failure and arrhythmias (Toepfer et al., 2020).

It is worth pointing out that similar to VIM and NPPA, the calcium sensor and modulator S100A6 is also upregulated in the heart of rats with ISO-iCM. Given that S100A6 is involved in the regulation of cytoskeleton and sarcomere structures by interacting with ANXA2, TAGLN, and MYL12a, which are all upregulated in the model rats, the elevated level of S100A6 may contribute to the disorder in cytoskeleton and cardiomyocyte motility *via* modulating the homeostasis of calcium ions (Breen and Tang, 2003; Nowotny et al., 2003).

Being a member of the myosin family and molecular motors of the sarcomere, Myosin 11 (MYH11) is a protein complex contributing to cell contraction by hydrolysis of ATP (Zhu et al., 2006). The mutation of the *Myh11* gene may cause familial thoracic aortic aneurysm and dissection heart, in which the aortic was enlarged near the heart (El-Hamamsy and Yacoub, 2009). There have been so far few reports on the relationship of MYL12a with heart-related diseases, but Park et al. (2011) found that this protein could influence cell morphology and dynamics by sustaining the stability of the Myosin 11. In *Myl12a* knockout cells, the MYH9, MYH10, and MYH6 expressions were significantly reduced. Taking together, our work suggests that MYH11 and MYH12a are also implicated in the development of ISO-iCM in rats in addition to other members, MYL4, MYH6/7, and MYL6L, of the myosin family.

Both PRPH and VIM belong to the type III intermediate filament family. PRPH is primarily expressed in peripheral neurons. The expression of *Prph* is upregulated during neuron development or regeneration when axons were injured (Hol and Capetanaki, 2017). With regard to this, the upregulation of PRPH in rats with ISO-iCM may suggest damage to the nervous system of the heart. VIM is mainly expressed in undifferentiated mesenchymal origin cells and regulates the functions of the lysosome, Golgi complex, and mitochondria organelles. Moreover, VIM can also influence the transportation of the integrins to the plasm membrane, and may then impact cell-extracellular matrix adhesion (Hol and Capetanaki, 2017). Taking this into account, the intermediate filament family proteins PRPH and VIM may be involved in the development of ISO-iCM in rats *via* damaging heart nerves and regulating cytoskeleton and myofibril structure.

Transgelin (TAGLN) is an actin filament binding protein and is also named smooth muscle protein 22-α (SM22-α). It was reported to be associated with tumorigenesis (Yu et al., 2013). Studies on patients with pulmonary arterial hypertension (PAH) in congenital heart disease (CHD) showed that TAGLN overexpression may promote the proliferation, migration, and cytoskeleton strengthening of pulmonary arterial smooth muscle cells (PASMCs) (Huang et al., 2018a). The upregulation of TAGLN in the heart of rats with ISO-iCM implicates its participation in cardiomyopathy by functioning as smooth muscle protein.

The membrane primary amine oxidase (AOC3) is an extracellular matrix protein. Although there is no evidence for direct association of AOC3 with heart failure, this protein has been identified as a new marker of myofibroblasts (Hsia et al., 2016). Hence, the protein is likely to be involved in the process of cardiomyocyte fibrosis and has a close relationship with ISO-iCM.

Two immune-related proteins, the Ig kappa chain C region (LOC500180) and BST2 were unraveled to be significantly upregulated and downregulated, respectively, in the heart of rats with ISO-iCM. A clinical study suggested that combined free light chains (cFLCs), which include both kappa and lambda FLCs, could be a marker of heart failure prognosis (Jackson et al., 2015). However, another report argued that a more mechanistic understanding of heart failure pathophysiology is needed to support the conclusion (Januzzi and Mann, 2015). Our results herein verified that Ig kappa chain C may play an important role in cardiomyopathy, perhaps *via* involvement in the inflammatory pathway (Kang et al., 2009). BST2 is the only protein that was downregulated in rats with ISO-iCM. It is an antiviral antigen implicated in the regulation of viral infection (Evans et al., 2010). Despite its antiviral functions, BST2 may be involved in some disease manifestations, for example, cancer and autoimmune diseases (Mahauad-Fernandez and Okeoma, 2016). Recently, the research found that circulating healing (CH) cells expressing BST2 are functionally activated by the injury-regulated systemic factor hepatocyte growth factor-activator (HGFA), which could participate in tissue repair (Lo Sicco et al., 2018). With regard to this, our finding that BST2 was downregulated in rats with ISO-iCM implies myocardial inflammatory damage in the rats.

The gene ontology (GO) enrichment showed that the most associated Kyoto Encyclopedia of Genes and Genomes (KEGG) pathways and biological process (BP) of the 25 DEPs are cardiac muscle contraction and muscle system process, respectively (Figure 6B). Since the most of the 25 DEPs, including eight extracellular matrix proteins and six myosin family proteins, and five actin-binding proteins, are involved in the cytoskeleton and myofibril structure and function, the disorders in cardiac muscle contraction, in particular structure and function of sarcomeric myosin motor, is most likely the main pathogenic elements of ISO-iCM in rats, similar to DCM and HCM in human.

The functional enrichment by STRING added 100 proteins (nodes) and 1,437 interactions (edges) to the initial 25 DEPs by matching the best interactor criteria (Supplementary Figure S6A). Notably, GO analysis annotated the most associated KEGG pathway and the biological process (GOBP) of the 125 proteins to be focal adhesion and muscle system process (Supplementary Figure S6B), again indicating that abnormality in structures of cytoskeleton and sarcomere are the characteristic phenotypes of ISO-iCM in rats.

**FIGURE 8:**
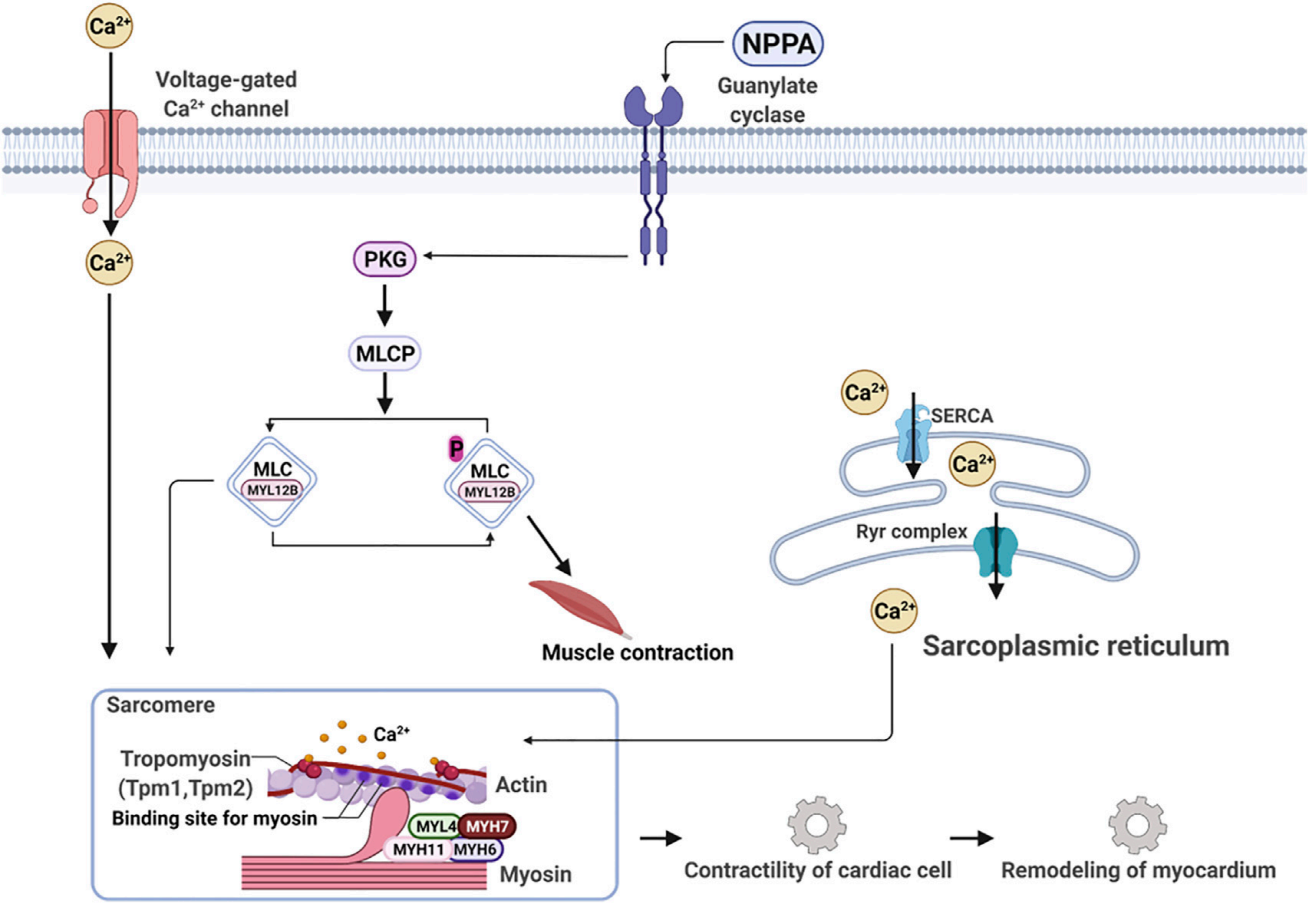
NPPA-triggered signaling pathway works together with the calcium signaling pathway to modulate contractility and remodeling of the myocardium.

Next, we applied the Ingenuity Pathway Analysis (IPA) program to enrich the core signaling pathways, which the 25 DEPs are associated with. The top 20 core signaling pathways of which the 25 DEPs are most associated with are depicted in Supplementary Figure S7. We can see that the myosins, for example, MYH6 and MYH7, and tropomyosins (TPM1 and TPM2) are deeply implicated in the dilated cardiomyopathy signaling pathway (Supplementary Figure S8) and the calcium signaling pathway (Supplementary Figure S9), of which both play a crucial role in the contractility of cardiac cell and the remodeling of the myocardium (Figure 8).

## Discussion

The occurrence and development of heart failure are closely related to the disorder of water and electrolyte metabolism (Chobanian et al., 1957), which are mainly modulated by natriuretic peptides A (NPPA) and GMP signaling (Moro and Lafontan, 2013; Song et al., 2015). In this work, we used isoproterenol to induce cardiomyopathy in rats. Our results indicated that isoproterenol is bound to β2-adrenergic receptor, and indeed upregulated NPPA expression by activating STA3 and GATA4 in the heart of rats. Meanwhile, the activation of STAT3 and GATA4 also promoted remarkably the expression of myosins MYH6, MYH7, MYH11, and MYL4, and tropomyosins TPM1 and TPM2. The myocardial contraction requires two proteins, myosin, and actin (Daniels et al., 2021). Sarcomeric myosin is the molecular engine of the heart, which converts the chemical energy of ATP into movement and regulates force production for contractibility of the whole heart. Myosin acts as a molecular accelerator or brake depending on whether and how it binds to actin. Actin is controlled by fluctuating calcium levels, which regulate a molecular clutch shielding actin *via* the troponin complex (TPM1 and TPM2) from myosin in a low diastolic Ca^2+^ level and exposing actin at a high systolic Ca^2+^ level (Daniels et al., 2021) to myosin binding, thereby activating myosin-ATPase activity to power heart movement. Therefore, any changes in ATP consumption and force production caused by genetic mutations on myosin and/or abnormal activation/suppression of myosin motor could lead to cardiac diseases, for example, hypertrophic cardiomyopathy (HCM) and dilated cardiomyopathy (DCM). With regard to this, several small molecule inhibitors and activators of myosin have been developed for treating inherited cardiomyopathies with genetic variants in myosin and entered different phases of clinic trails (Lehman et al., 2022). The drug candidates, for example, mavacamten, were demonstrated to have a spatially distinct effect in the sarcomere and are thought to be root-targeting therapeutics without harmfulness to other organs (Lehman et al., 2022).

Our quantitative proteomics data demonstrated that apart from about 12-fold increase in the NPPA level, the expression levels of myosin family proteins, MYL4, MYH6, MYH7, MYL12A, MYL6L, and MYH11, and actin filament binding proteins TAGLN, TPM1, and TPM2, were all elevated in the heart of the model rats with ISO-iCM. This remarkably indicates the upregulation of myosin-ATPase activity. Taking the increase in the expression of calcium sensor S100A6 into account, the model rats appeared to have similar characteristics to the inherited HCM, in which the genetic variants in sarcomeric myosin and myofilament increase the proportion of active myosin, that is, more accelerator and less brake, leading to diastolic dysfunction and hypercontractility (Daniels et al., 2021). Also, thanks to the precise MS quantification of proteins, we demonstrated that DS significantly reversed the expression of the myosin family proteins and actin filament binding proteins, except for MYH6 and MYH7, showing a good pharmacological effect on ISO-iCM in rats. More importantly, our study revealed that the major active component AS4 in AM remarkably improved the pharmacological efficiency of DS on ISO-iCM in rats by complementarily reversing the expression of MYH6 and MYH7, and further downregulating NPPA, MYK4, and PAM. These results strongly suggest that the combination of DS with AS4 exerted pharmacological benefits on ISO-iCM in rats by synergistically modulating the myosin motor. Another active component calycosin-7-glucoside (C7G) was also shown to improve the pharmacological efficacy of DS on ISO-iCM. However, the improvement was less than AS4, most likely due to the weaker ability of C7G to downregulate NPPA, MYL4, and PAM.

As mentioned earlier, AM is often used in combination with DS. However, in the present work, we did not find that the combination of DS and AM produced a pronouncedly higher pharmacological potential for ISO-iCM in rats than the use of DS alone. We have also investigated the pharmacological efficiency of AM used alone and found that the pharmacological effect of AM alone on ISO-iCM in rats was poor (data now showed). To sort out why the combination of DS and AM could not provide better pharmacological benefits on ISO-iCM, we further studied the effect of Astragalus polysaccharides (APS) on the pharmacological efficiency of DS. Surprisingly, we found that APS upregulated NPPA, MYL4, and PAM, which are the most contributors to the development of ISO-iCM in rats, thereby reducing the pharmacological efficiency of DS on ISO-iCM. This suggests that it is the presence of APS in AM that reduced the pharmacological effect of DS combined with AM on ISO-iCM in rats. These findings imply that the combination of TCMs is highly necessary, but that the simple combination may not produce expected outcomes.

On the other hand, our quantitative proteomics results showed that DS decoction significantly upregulated the expression of MYH7, which as mentioned earlier plays a vital role in development of cardiomyopathy, and downregulated the expression of BDH1, which acts as an actin filament binding protein and is involved in cardiac diseases (Brahma et al., 2020). However, we herein did not identify which components in DS caused the differential expression of these two proteins. To address this issue, it is necessary to perform the proteomics analysis after treating the ISO-iCM model rats with purified active components, for example, quercetin and kaempferol, from DS. This work will be undertaken in the near future in our laboratory.

## Conclusion

In this work, with the use of quantitative mass spectrometric proteomics analysis, we revealed for the first time that the classic traditional Chinese medicine, *Descurainia sophia* seeds (DS) relieved isoproterenol-induced cardiomyopathy (ISO-iCM) in rats by reversing the most of differentially expressed proteins (DEPs), including molecular motor MYL4 and the well-known biomarker NPPA of cardiomyopathy. Importantly, we demonstrated that the combined use of DS with *Astragalus mongholicus* (AM) did not provide better pharmacological benefit to ISO-iCM, while astragaloside IV (AS4), a major active component in AM, significantly improved the pharmacological effect of DS on ISO-iCM *via* complementarily or further reversing myosins MYH6/7, and NPPA and MYL4. This suggests that DS and AS4 synergistically work on modulating the myosin motor in sarcomere to achieve better pharmacological benefit to cardiomyopathy. In contrast, the combination of DS with *Astragalus* polysaccharides (APS) from AM even reduced the pharmacological efficiency of DS to ISO-iCM in rats, most likely contributing to the less improvement of AM on the pharmacological efficiency of DS*.* This work highlights the power of mass spectrometric proteomics strategy combined with conventional pathological approaches for the development and modernization of TCM. Significantly, our findings provide novel insights to better understanding and improving the combination pharmacological principle of traditional Chinese medicine.

## Data Availability

The datasets of proteomics analysis presented in this study can be found in online Proteomics Identification Database at ebi.ac.uk/pride/ with a reference number of 1-20220509-37752.
