# Supplementary material for: Astragaloside IV promotes pharmacological effect of Descurainia sophia seeds on isoproterenol-induced cardiomyopathy in rats by synergistically modulating the myosin motor
Source: Front Pharmacol. 2022 Aug 11;13:939483. doi: 10.3389/fphar.2022.939483 (PMC9403516; doi:10.3389/fphar.2022.939483)
Supplement: Supplementary file 3 [file DataSheet1.PDF]

Astragaloside IV Promotes Pharmacological Effect of *Descurainia sophia* seeds on Isoproterenol-Induced Cardiomyopathy in Rats by Complementarily Modulating Myosin Motor

Xingkai Liu[1,4†], Qian Chen[2†], Xuming Ji[3†], Wanchen Yu[2], Tong Wang[2], Juanjuan Han[1], Shumu Li[1], Jianan Liu[1], Fangang Zeng[5], Yao Zhao[1], Yanyan Zhang[1], Qun Luo[1,4*], Shijun Wang[2*], Fuyi Wang[1,2,4*]

[1] Beijing National Laboratory for Molecular Sciences; CAS Research/Education Center for Excellence in Molecular Sciences; National Centre for Mass Spectrometry in Beijing; CAS Key Laboratory of Analytical Chemistry for Living Biosystems, Institute of Chemistry, Chinese Academy of Sciences, Beijing, 100190, P. R. China;

[2] College of Traditional Chinese Medicine, Shandong University of Traditional Chinese Medicine, Jinan, 250355, P.R. China;

[3] Academy of Chinese Medical Science, School of Basic Medical Science, Zhejiang Chinese Medical University, Hangzhou, 310053, P.R. China;

[4] University of Chinese Academy of Sciences, Beijing 100049, P.R. China

[5] School of Environment and Natural Resources, Renmin University of China, Beijing, 100872, P. R. China

[†]These authors have contributed equally to this work

*** Correspondence:** Fuyi Wang(fuyi.wang@iccas.ac.cn); Shi-jun Wang (wsj@sdutcm.edu.cn); Qun Luo (qunluo@iccas.ac.cn)

Supplementary Materials

Supplementary Tables S1 – S5

Supplementary Figure S1 – S9

Supplementary Table S1. The parameters of MS/MS analysis.

Ion source spray voltage:	2200V		
Sweep Gas (Arb):	0		
Ion transfer tube temp:	320℃		
Master scan:		Data dependent MS scan:	
Detector type:	orbitrap	Isolation mode:	quadrupole
Orbitrap resolution:	60000	Activation type:	HCD
Scan range:	350-1800	Collision energy:	40%
RF Lens (%):	30	Detector type:	orbitrap
AGC target:	4.00E+05	Resolution:	30000
Maximum injection time (ms):	50	First mass (m/z):	110
Microscan:	1	AGC Target:	5.00E+04
Monoisotopic peak determination:	peptide	Inject ions for all available parallelizable time:	TRUE
Include charge state(s):	2-7		
Exclusion duration(s):	20		
Mass tolerance:	+20ppm		
Intensity threshold:	2.00E+04		
Data dependent mode:	cycle time		
Time between master scans(s):	3		

Supplementary Table S2: Compounds identified commonly in *Descurainia Sophia* seed decoction and *Astragalus mongholicus* decoction by UPLC-MS/Ms.

Supplementary Table S3: Compounds identified exclusively in *Descurainia Sophia* seed decoction by ESI-MS/MS.

Supplementary Table S4: Compounds identified exclusively in *Astragalus mongholicus* decoction.by ESI-MS/MS

Supplementary Table S5: MS/MS raw data for proteins identification and quantification in the heart of rats with isoproterenol-induced cardiomyopathy before and after treatments.

These tables are separately provided in Excel format.

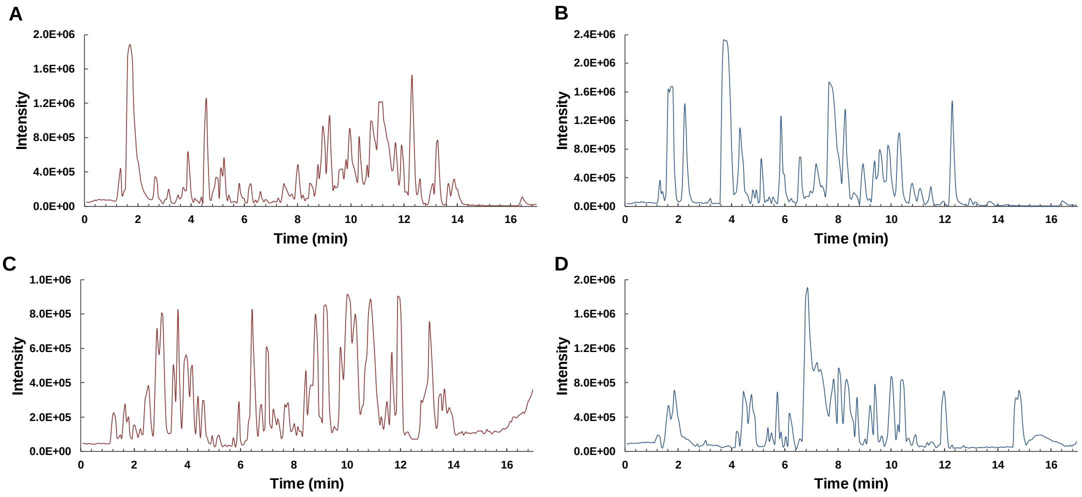

Supplementary Figure S1. Total ion count (TIC) chromatograms of (A, B) *Descurainia Sophia* seed decoction, and (C, D) *Astragalus mongholicus* decoction detected by mass spectrometry under positive ion mode (A, C) and negative ion mode (B, D), respectively.

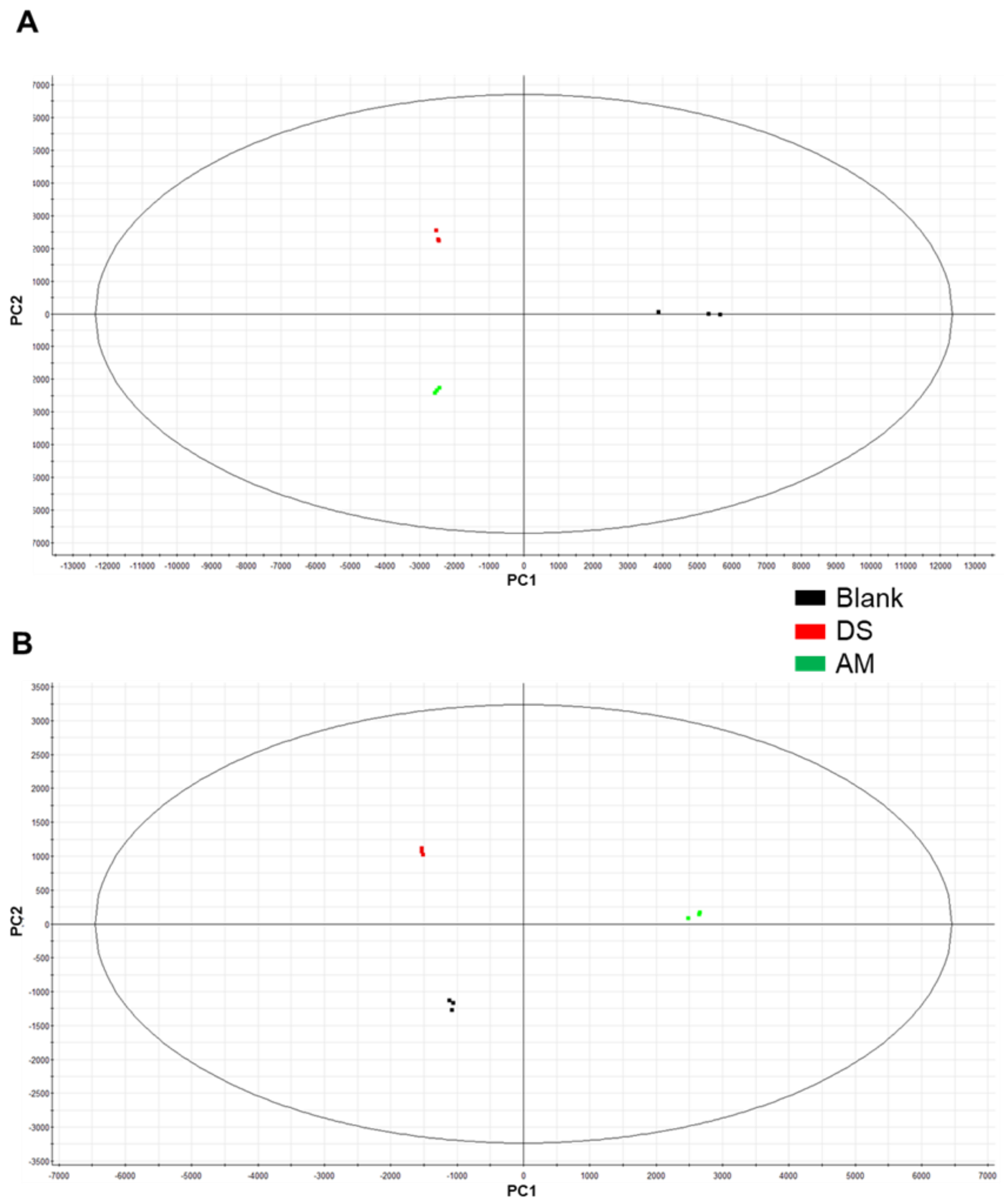

Supplementary Figure S2. PCA results based on the positive (A) and negative (B) ESI-MS spectra of *Descurainia Sophia* seed (DS) and *Astragalus mongholicus* (AM) decoctions shown in Figure S1.

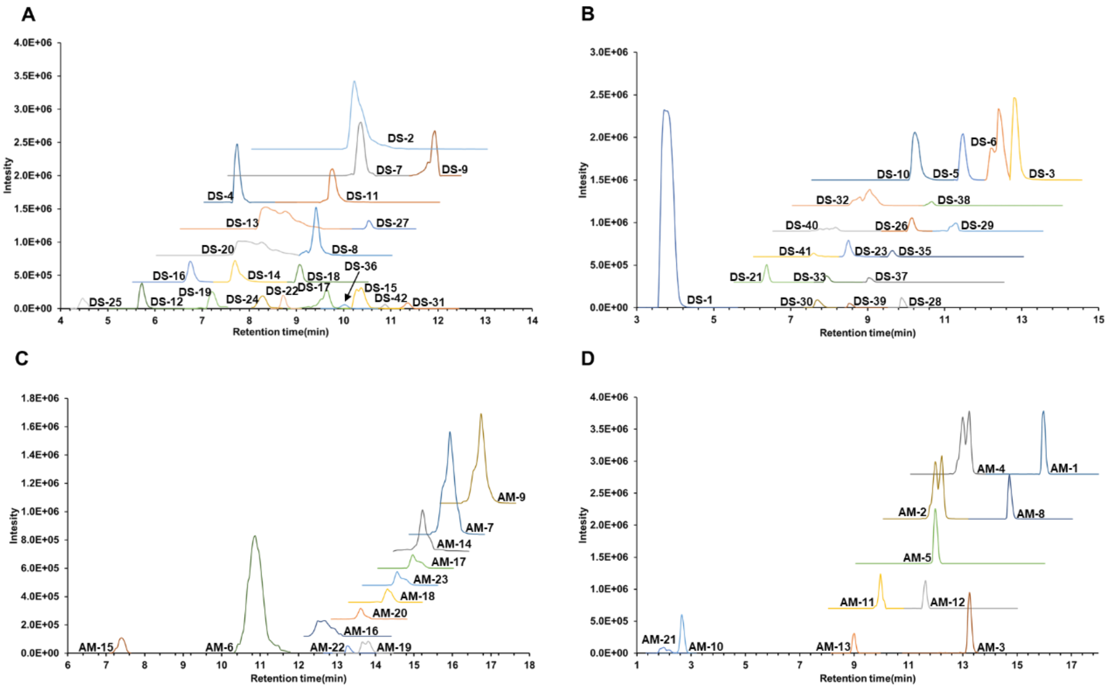

Supplementary Figure S3. Extracted ion count (EIC) chromatograms of (A, B) *Descurainia Sophia* seed decoction, and (C, D) *Astragalus mongholicus* decoction detected by mass spectrometry under positive ion mode (A, C) and negative ion mode (B D), respectively. The detailed information of the compounds identified based on these peaks are listed in Tables 1 and 2.

A

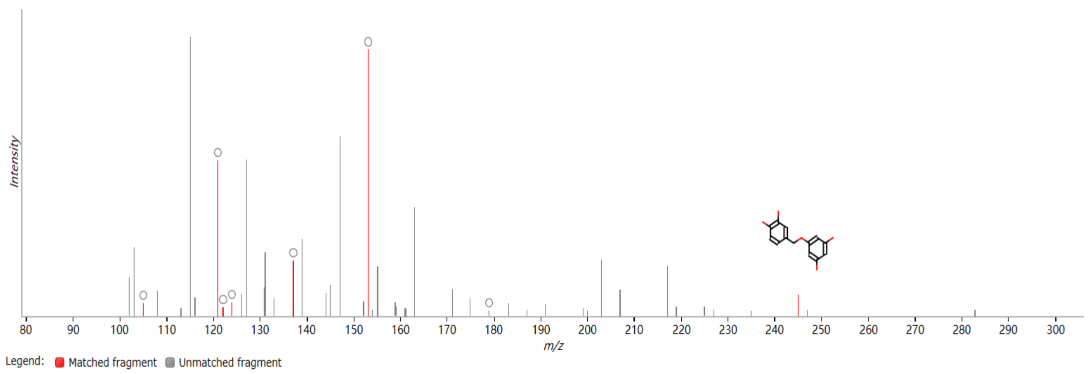

B

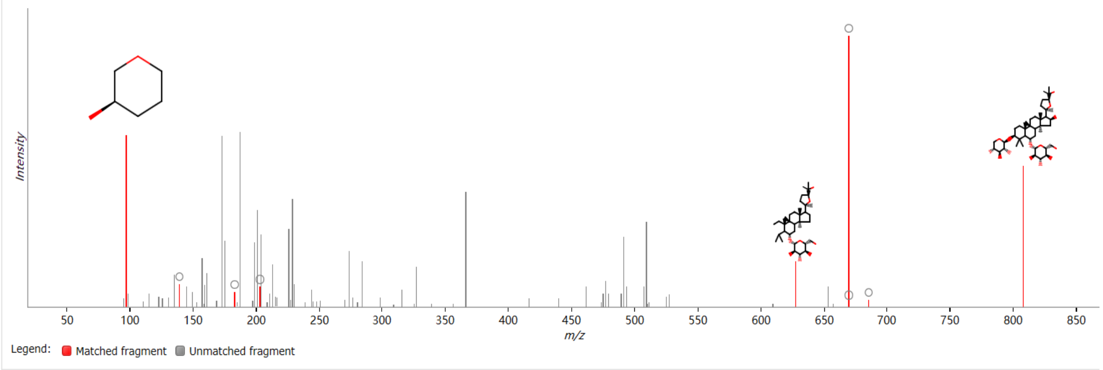

Supplementary Figure S4. MS/MS spectra of (A) quercetin and (B) Astragaloside IV.

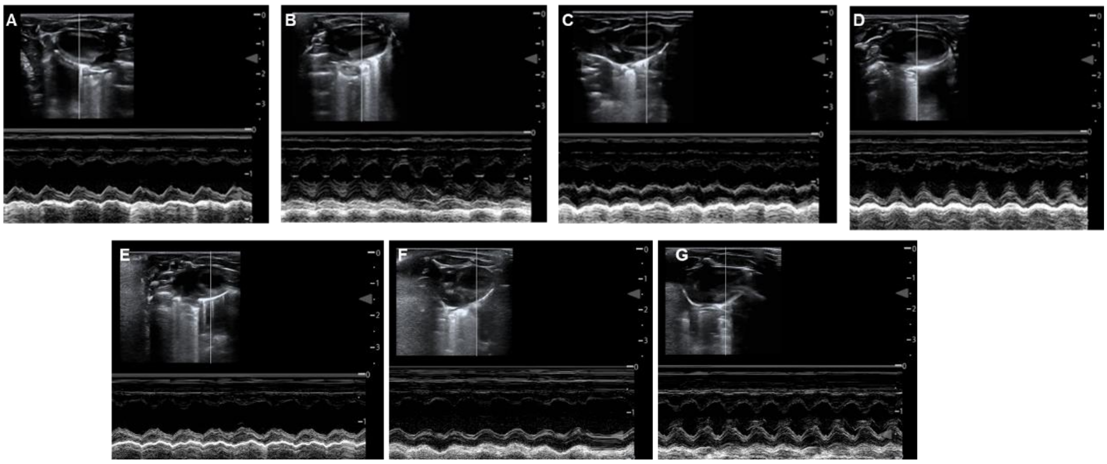

Supplementary Figure S5. Representative echocardiographics of rats in different groups. (A) Control group (CM); (B) model group (MG); and (C - F) treated groups with (C) *Descurainia Sophia* seed decoction alone (SL), and (D) *Descurainia Sophia* seed plus *Astragalus mongholicus* decoction (SLAM), (E) *Descurainia Sophia* seed decoction plus astragaloside IV (SLAS4), (F) calycosin-7-glucoside (SLC7G), or (G) astragalus polysaccharides (SLAPS). The echocardiographic data were used to calculate the left ventricular ejection fraction (LVEF) and left ventricular fraction shortening (LVFS) shown in Figure 3A-B.

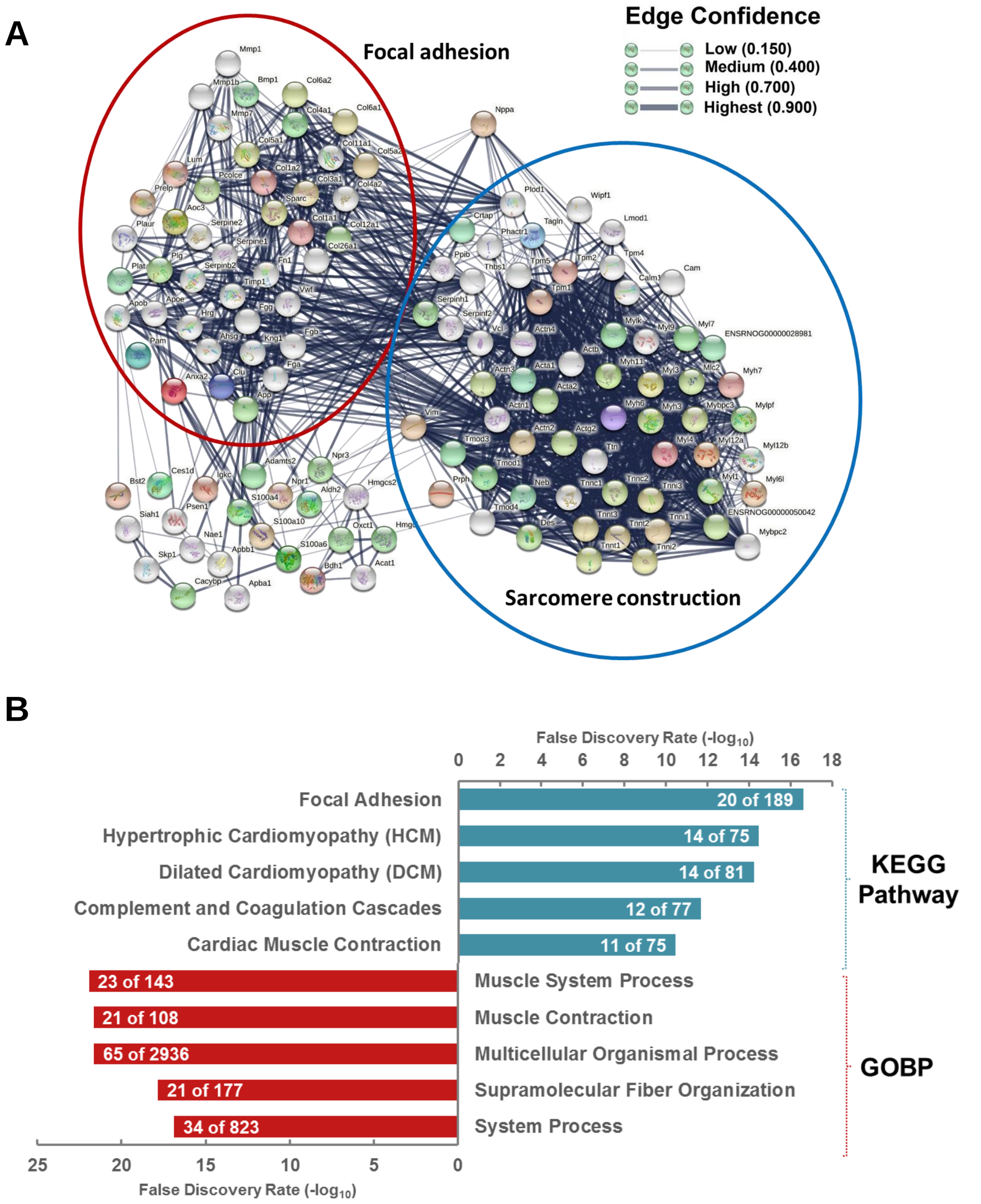

Supplementary Figure S6. GO annotation of KEGG pathway and biological process (GOBP) of differentially expressed proteins (DEPs) in the heart of rats with Isoproterenol-Induced Cardiomyopathy compared to the controls. (A) The protein-protein interaction network after addition of 100 protein to the initial 25 DEPs by matching the best interactor criteria. (B) KEGG pathways and biological processes which the 125 proteins are associated with.

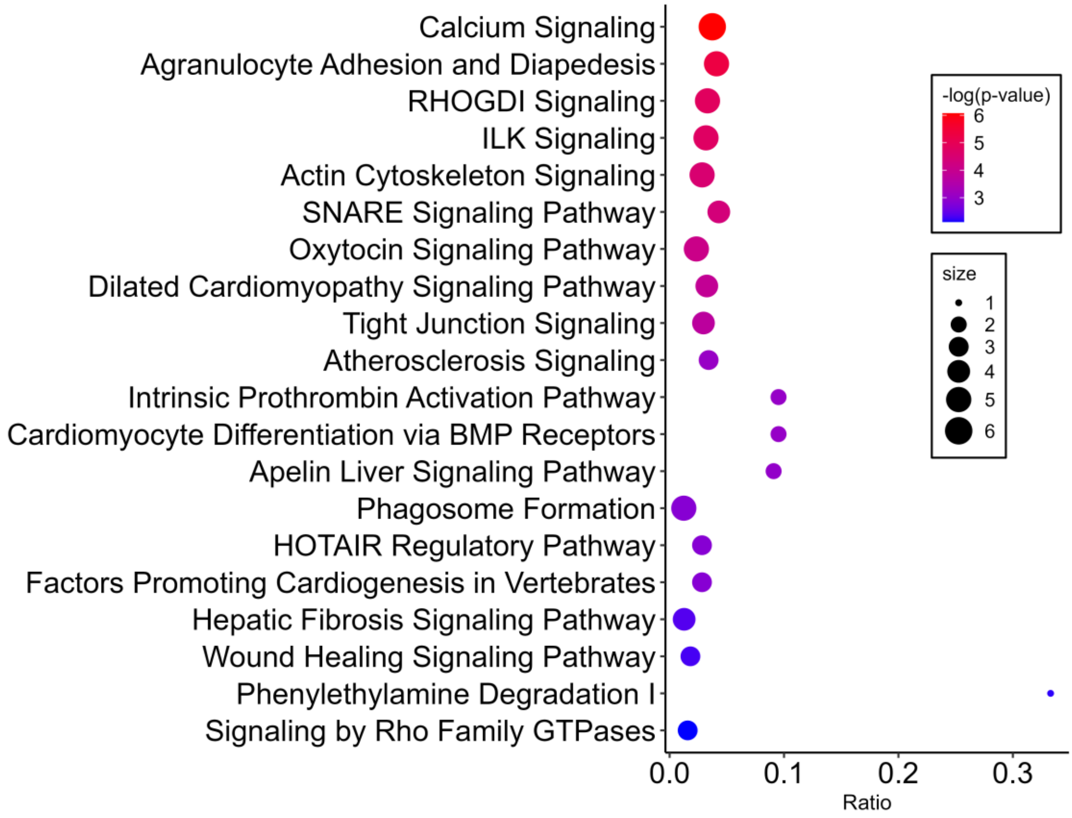

Figure S7. Top 20 core signaling pathways with which the 25 DEPs identified in the heart of rats with ISO-iCM are highly associated. The higher the value of –log(*P*) for a signaling pathway, the more closely the DEPs are associated with it.

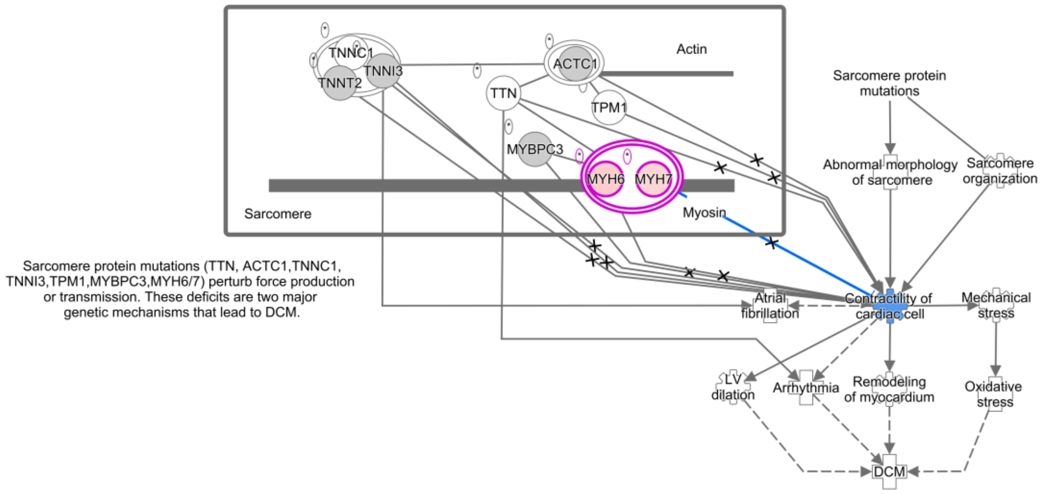

Figure S8. The dilated cardiomyopathy signaling pathway which myosin (MYH6 and MYH7) and tropomyosin TPM1 are highly associated with.

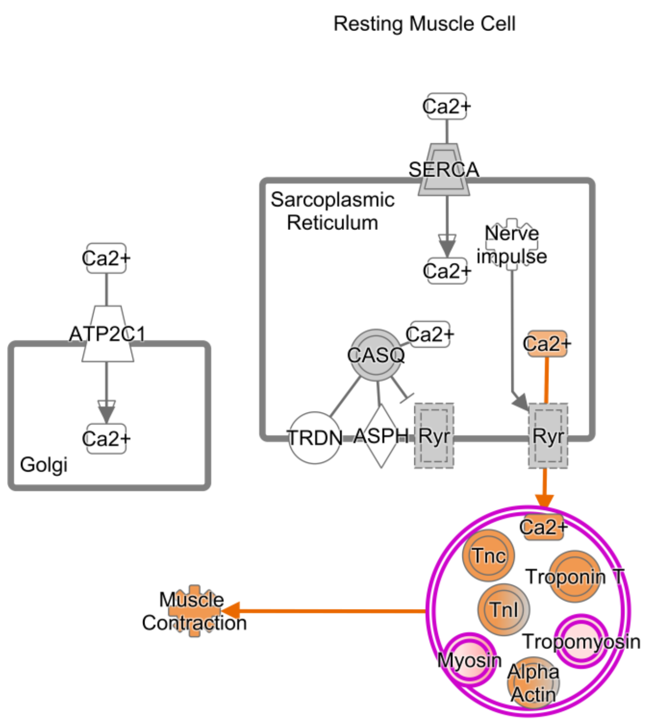

Figure S9. The calcium signaling pathway which myosin and tropomyosin complexes are highly associated with.
